# Enhancing Capacity and Stability of Anionic MOFs as Electrode Material by Cation Exchange

**DOI:** 10.3389/fchem.2022.836325

**Published:** 2022-03-04

**Authors:** Oluseun Akintola, Patrick Gerlach, Christian T. Plass, Andrea Balducci, Winfried Plass

**Affiliations:** ^1^ Institut für Anorganische und Analytische Chemie, Friedrich-Schiller-Universität Jena, Jena, Germany; ^2^ Institut für Technische Chemie und Umweltchemie, Friedrich-Schiller-Universität Jena, Jena, Germany; ^3^ Center for Energy and Environmental Chemistry Jena (CEEC Jena), Jena, Germany; ^4^ Institut für Festkörperphysik, Friedrich-Schiller-Universität Jena, Jena, Germany

**Keywords:** anionic-MOF, cation exchange, lithium, sodium, insertion, batteries

## Abstract

In this study we report on the characterization and use of the anionic metal-organic framework (MOF) JUMP-1, [(Me_2_NH_2_)_2_[Co_3_(ntb)_2_(bdc)]]_*n*_, alongside with its alkali-metal ion-exchanged analogs JUMP-1(Li) and JUMP-1(Na), as electrode materials for lithium and sodium batteries. Composite electrodes containing these anionic-MOFs were prepared and tested in 1 M lithium bis(trifluoromethylsulfonyl)imide (LiTFSI) in propylene carbonate (PC) and/or 1 M sodium TFSI (NaTFSI) in PC. We showed that the ion-exchanged materials JUMP-1(Li) and JUMP-1(Na) display higher capacities in comparison with the original as-prepared compound JUMP-1 (490 mA∙h∙g^−1^ vs. 164 mA∙h∙g^−1^ and 83 mA∙h∙g^−1^ vs. 73 mA∙h∙g^−1^ in Li and Na based electrolytes, respectively). Additionally, we showed that the stability of the electrodes containing the ion-exchanged materials is higher than that of JUMP-1, suggesting a form of chemical pre-alkalation works to stabilize them prior to cycling. The results of these studies indicate that the use of designed anionic-MOFs represents a promising strategy for the realization of high performance electrodes suitable for energy storage devices.

## 1 Introduction

One of the most important successes of the last decades in the area of energy storage is the development of rechargeable lithium-ion batteries (LIBs). Commercial LIBs display high energy density (250 W∙h∙kg^−1^) and high cycling stability and are nowadays used in a large number of applications, ranging from power sources in electronic devices to electric vehicles (Etacheri et al., 2011; Goodenough and Park, 2013; Manthiram, 2017; Li et al., 2018). The state-of-the-art LIBs contain cathodes based on metal oxides, e.g., NMC (Nickel manganese cobalt oxide), and anodes based on graphite. This latter material is used because it displays a relatively high capacity (372 mA∙h∙g^−1^), low lithiation potential, and is low in cost (Winter et al., 2018). In the last years, however, several efforts have been made towards the development of alternative anodic materials that are able to display higher specific capacity compared to graphite. With this aim, several carbonaceous materials, e.g., graphene and carbon fiber have been considered (Noel and Suryanarayanan, 2002; Chen et al., 2009; Guo et al., 2009; Kumar et al., 2009; Lian et al., 2010; Sun et al., 2018; Yoshino, 2021). Furthermore, several non-carbonaceous compounds have likewise been proposed and tested (Li et al., 2020; Yang et al., 2020). Among them, one interesting group of compounds are metal–organic frameworks (MOFs) (Zhao et al., 2018; Shrivastav et al., 2019).

MOFs are porous materials that belong to the continuously growing class of polymeric coordination compounds, whose potential applications range from the well-known gas storage and separation (Han et al., 2009; Murray et al., 2009; Suh et al., 2012), catalysis (Lee et al., 2009; Ma et al., 2009; Dhakshinamoorthy and Garcia, 2012), magnetism (Kurmoo, 2009; Dechambenoit and Long, 2011; Weng et al., 2011; Campo et al., 2016), drug delivery (Horcajada et al., 2008; Horcajada et al., 2010; Sun et al., 2013) and sensing (Xiao et al., 2010; Kreno et al., 2012) to the lesser known utilizations as spin qubits (Yamabayashi et al., 2018; Jellen et al., 2020; Yu et al., 2020). The general motif of their structures typically involves a polytopic organic ligand that is coordinatively linked to transition metal centers or clusters *via* donor atoms such as oxygen or nitrogen (Cook et al., 2013; Ghasempour et al., 2021).

In recent years, different types of MOFs have also been investigated as electrode materials for several energy storage devices, including LIBs and sodium-ion batteries (SIBs), showing promising performance while delivering high capacities (Maiti et al., 2015; Hu et al., 2016; Weng et al., 2020; Baskoro et al., 2021; Feng and Wen, 2021). Many of these compounds incorporate transition metal ions such as Mn(II), Co(II), Ni(II), and Cu(II) (Zhang et al., 2014; Calbo et al., 2019; Wang et al., 2020). A good number of these MOFs are known to contain mostly aromatic ligands incorporating a variety of donor groups ranging from oxygen-based carboxylates (Gou et al., 2014; Dong and Xu, 2017) to nitrogen-based groups such as pyridine (Tian et al., 2016) and imidazole and even rare examples such as thiolate groups (Wu et al., 2020). These functional groups not only play structural roles as points of linkages, but also provide the ability to interact with additional cations within the pores, allowing reversible insertion of lithium and sodium ions (Yang et al., 2009; Hu et al., 2017; Ning et al., 2017).

While the vast majority of MOFs tend to be neutral, anionic networks have also been known to occur. Such anionic networks are usually obtained under synthetic conditions in which the solvent DMF or DEF tends to degrade, resulting in the formation of dimethylammonium or diethylammonium cations, which in turn template the formation of anionic networks, while remaining as guests within the pores (Burrows et al., 2005). Notwithstanding this fact, anionic frameworks are clearly an interesting subclass of MOFs, particularly in that they offer the advantage of tuning their pore sizes via post-synthetic cation exchange (Yang et al., 2008; Procopio et al., 2010; Akintola et al., 2017a; Liu et al., 2017). A further positive consequence of the negatively charged nature of the framework is the abundance of electronegative sites that enable ion mobility within the channels (Duan et al., 2021), which has been exploited in proton conductivity applications (Liu et al., 2016) and could play a role in making them more amenable for the use as electrodes in a similar way. Furthermore, pre-alkalation of the MOF with the target inorganic cations *via* cation exchange could provide a path to improving their performance by preparing the materials for insertion.

**FIGURE 1:**
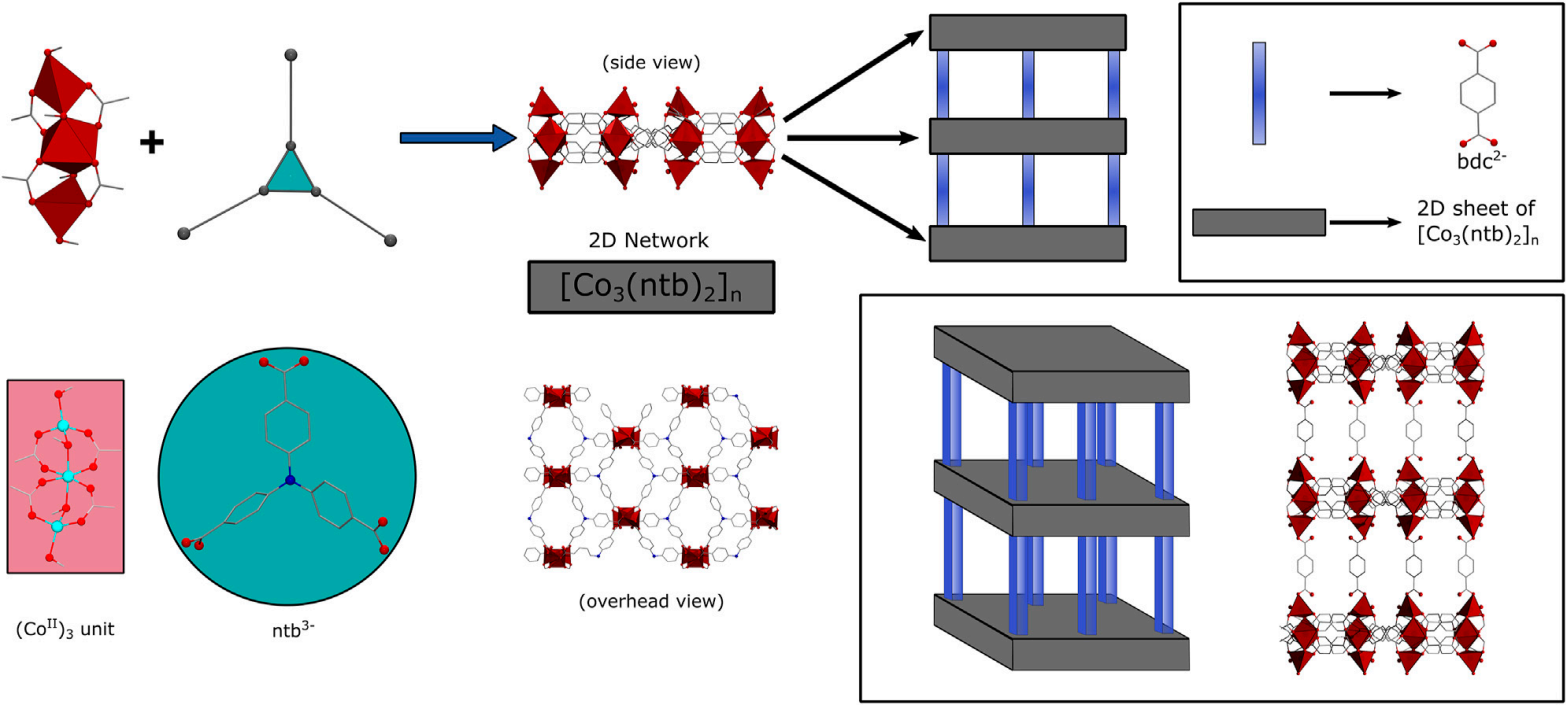
Schematic representation of the basic construction of JUMP-1: 2D layers are generated from trinuclear cobalt (II) clusters and nitrilotribenzoic acid anions (ntb^3-^) and further assembled with terephthalic acid anions (bdc^2-^) as pillars to the overall three-dimensional architecture.

Herein, we report the use of JUMP-1 (Akintola et al., 2017b) and its lithium- and sodium-exchanged analogs [further denoted as JUMP-1(Li) and JUMP-1(Na)] as anodic material for LIBs and SIBs. JUMP-1 is a so-called pillared-layer MOF with an anionic framework, which is composed of neutral two-dimensional (2D) networks linked by terephthalic acid as pillared liners. The 2D networks are derived from the redox-active nitrilotribenzoic acid as organic linker and trinuclear cobalt (II) clusters as inorganic nodes. A schematic representation of the structure of JUMP-1 is depicted in Figure 1. The electrochemical behavior of JUMP-1 and its ion-exchanged materials JUMP-1(Li) and JUMP-1(Na) will be compared with the aim to understand whether the cation exchange prior to the electrochemical usage has a positive impact on the electrochemical behavior of the electrodes.

## 2 Results and Discussion

### 2.1 Synthesis and Characterization of MOF Materials

**FIGURE 2:**
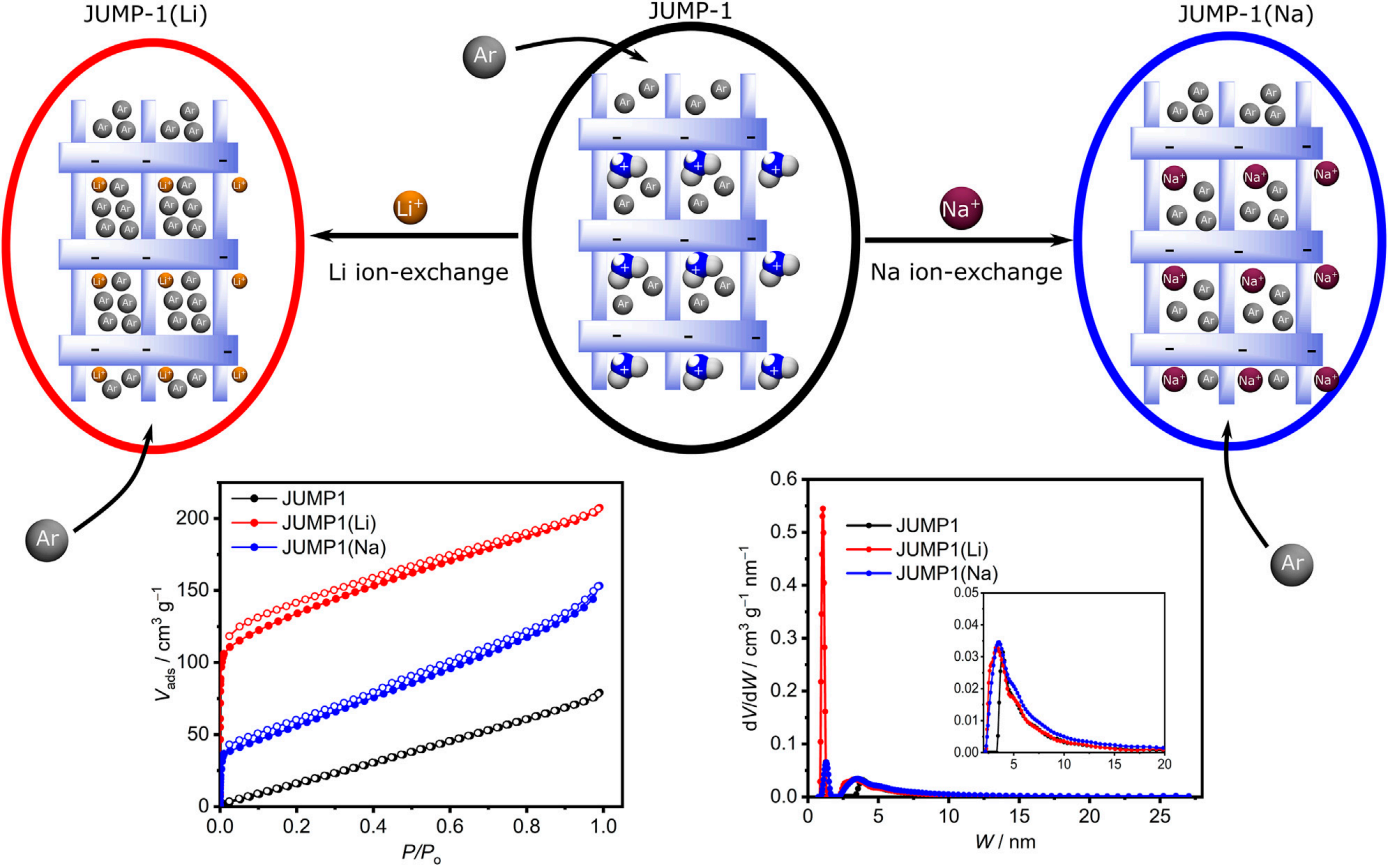
Top row: Schematic depiction of replacement of the dimethylammonium cations in JUMP-1 (center) by lithium and sodium cations to yield JUMP-1(Li) (left) and JUMP-1(Na) (right), respectively. The variation in the accessible pore volume is indicated by different number of Ar atoms depicted. Bottom row: Corresponding Ar adsorption isotherms (left) and pore size distributions (see text for details).

JUMP-1 with the formula [(Me_2_NH_2_)_2_[Co_3_(ntb)_2_(bdc)]]_*n*_ was synthesized under hydrothermal conditions utilizing a mixture of the tritopic ligand nitrilotribenzoic acid (H_3_ntb), terephthalic acid (H_2_bdc), and cobalt (II) chloride in DMF (Akintola et al., 2017b). The three-dimensional (3D) framework of JUMP-1 is anionic and its charge is balanced by two molecules of dimethylammonium per [Co_3_(ntb)_2_] repeating unit of the compound. These organic cations are located within the pore structure of the anionic framework. Replacement of the organic cation with lithium and sodium ions can be achieved by simply immersing the as-prepared material in saturated nitrate solutions of the respective ions in DMF and resulted in the cation-exchanged networks, JUMP-1(Li) and JUMP-1(Na), respectively (Figure 2). In order to increase the accessibility of the pores within the MOF structures, all three as-synthesized materials were activated through immersing them in ethanol and subsequent drying using supercritical CO_2_ prior to their further use (Akintola et al., 2021).

The elemental composition of JUMP-1 and its ion-exchanged analogs JUMP-1(Li) and JUMP-1(Na) were determined by CHN analysis. In addition, the replacement of the organic cation in the latter networks was ascertained by thermogravimetry and confirmed by the disappearance of the signature dip around 280 °C in the DTG traces, which is characteristic for the dimethylammonium ions present in JUMP-1 (Supplementary Figure S1). The stability of the network structure after the cation exchange and the crystallinity of the samples was probed by powder X-ray diffraction (PXRD), as depicted in the Supplementary Figure S2. For JUMP-1(Li), not only is the crystallinity of the sample confirmed, but also, in particular, that the framework retains its original structure. In the case of JUMP-1(Na), the PXRD clearly shows the crystallinity of the sample, although a variation in the peak pattern suggests changes in the overall structure that cannot be assigned to any specific molecular feature in the framework. However, based to the even increased porosity of JUMP-1(Na) with respect to JUMP-1 (vide infra), we assume that the basic framework structure is also present in JUMP-1(Na).

The porosity of the anionic framework JUMP-1 and its ion-exchanged analogs JUMP-1(Li) and JUMP-1(Na) was investigated by measuring Ar adsorption isotherms at 87 K for the activated materials (Figure 2). Within the series of the anionic frameworks JUMP-1, JUMP-1(Na), and JUMP-1(Li) an increasing surface area of 100, 180, and 420 g∙mol^−1^, respectively, is observed. This nicely corresponds with the decreasing size of the relevant counter cation present in the pores. Moreover, the analysis of the pore size distribution depicted in Figure 2 shows that the lithium-exchanged analog JUMP-1(Li) has the highest proportion of micropores in comparison with the other materials. This is consistent with the improved access stemming from a reduced steric demand as the size of the counter ion decreased.

### 2.2 Electrochemical Studies

In the following, the activated forms of the anionic framework JUMP-1 and its ion-exchanged analogs JUMP-1(Li) and JUMP-1(Na) were used for the preparation of the electrodes employed in the electrochemical studies. At first cyclic voltammograms (CVs) and galvanostatic charge-discharge profiles were measured, followed by a rate capability test and a long-term test for cycling stability with 1000 charge-discharge cycles.

#### 2.2.1 MOFs as Anodic Materials for LIBs

**FIGURE 3:**
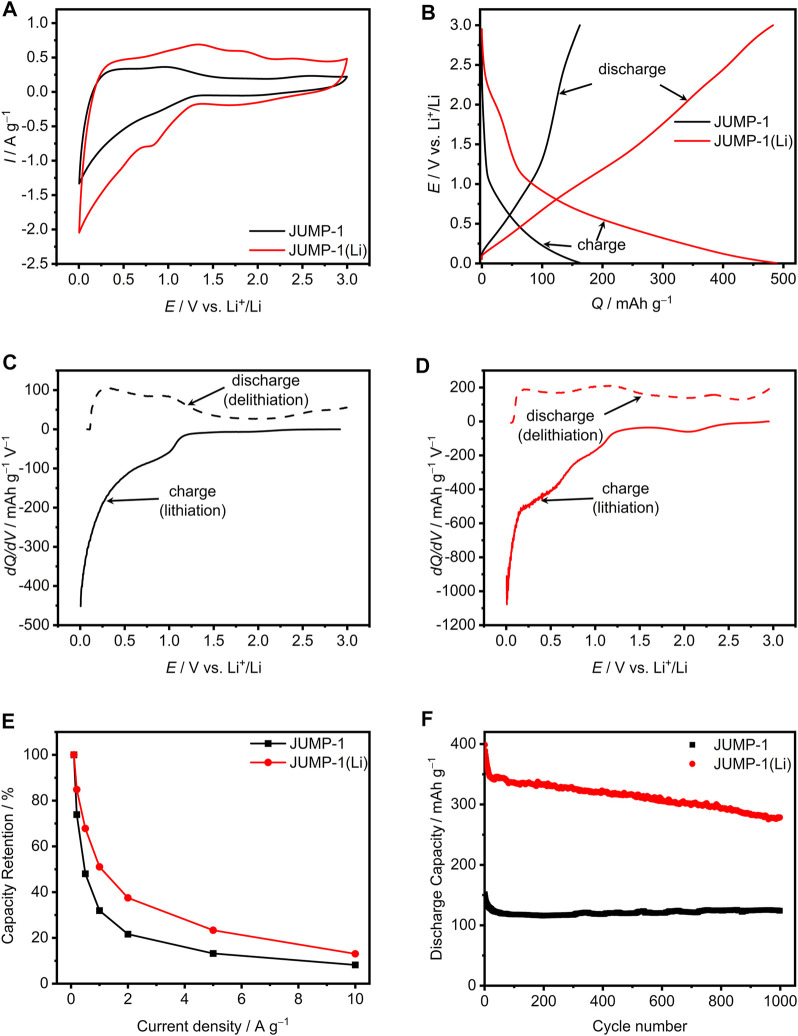
**(A)** CVs at 1 mV∙s^−1^ for JUMP-1 and JUMP-1(Li) in 1 M LiTFSI in PC. **(B)** Galvanostatic charge-discharge profiles at 1 A∙g^−1^ of JUMP-1 and JUMP-1(Li) in 1 M LiTFSI in PC. Differential capacity plots at 1 A∙g^−1^ for JUMP-1 **(C)** and JUMP-1(Li) **(D)** in 1 M LiTFSI in PC. **(E)** Rate capability from 0.1 to 10 A∙g^−1^ of JUMP-1 and JUMP-1(Li) in 1 M LiTFSI in PC. **(F)** Cycling stability at 1 A∙g^−1^ of JUMP-1 and JUMP-1(Li) in 1 M LiTFSI in PC.

The electrodes based on JUMP-1 and JUMP-1(Li) were investigated using 1 M lithium bis(trifluoromethylsulfonyl)imide (LiTFSI) in propylene carbonate (PC) as electrolyte. At first, impedance spectra of both electrodes have been measured. There the electrode of JUMP-1(Li) displays a higher initial charge transfer resistance in impedance spectra compared to JUMP-1 (Supplementary Figure S3A). The CVs of JUMP-1 and JUMP-1(Li) obtained at 1 mV∙s^−1^ are depicted in Figure 3A. As shown, although the two electrodes display rather comparable profiles, a substantial difference in term of measured current densities was observed. Specifically, JUMP-1(Li) displays significantly higher values of current through the entire voltage range compared to JUMP-1. This difference could be associated with the larger number of accessible sites within the pores present in the lithium-ion exchanged MOF.

Figure 3B displays a comparison of the galvanostatic charge-discharge profiles of the investigated MOFs materials at a current density of 1 A∙g^−1^. For both electrodes a rather sloping charge-discharge profile over the whole voltage range (from 0.005 to 3 V vs. Li^+^/Li) is observed. However, the capacity delivered by the two electrodes is significantly different. As a matter of fact, while the electrode with the MOF JUMP-1 displays a capacity of 164 mA∙h∙g^−1^ the electrode containing the pre-treated JUMP-1(Li) displays a capacity of 490 mA∙h∙g^−1^. This significant difference in terms of specific capacity indicates the occurrence of a higher insertion degree of lithium cations in the pre-treated ion-exchanged MOF JUMP-1(Li). Therewith it is evident, that with the pre-treatment a more suitable host structure for the insertion and release of lithium cations is generated.

In order to gain a better understanding of the different faradaic storage behaviors of JUMP-1 and JUMP-1(Li), Figures 3C,D compare the differential capacity curves of both electrodes, which were calculated from the galvanostatic charge-discharge profiles shown in Figure 3B. As displayed in Figure 3C, JUMP-1 in 1 M LiTFSI in PC provides charge storage over the complete potential range from 0.005 to 3 V vs. Li^+^/Li. However, the most pronounced faradaic processes were found between 0.005 and 1.25 V vs. Li^+^/Li. In particular for lithium insertion (full line, charge) peaks are observed at 1 V vs. Li^+^/Li and most prominent at the very low potential range near 0.005 V vs. Li^+^/Li. For the inverted process, i.e., the lithium release from the MOF material (broken line, discharge), peaks are present at 0.3 and 1 V vs. Li^+^/Li. The integrated areas of the lithium ion insertion and release, which can be understood as the total amount of charge stored or delivered in the charge and discharge process, are equal to 164 mA∙h∙g^−1^ and 162 mA∙h∙g^−1^, respectively, which results to a charge-discharge efficiency of 99%.

A similar behavior is found for JUMP-1(Li), as depicted in Figure 3D, with faradaic processes observed throughout the whole potential range between 0.005 and 3 V vs. Li^+^/Li. However, the total amount of charge stored and delivered for JUMP-1(Li) is 491 mA∙h∙g^−1^ for lithium insertion and 483 mA∙h∙g^−1^ for lithium release, which is much higher than that observed for JUMP-1. This is consistent with the findings related to the CVs (Figure 3A) and galvanostatic charge-discharge profiles (Figure 3B) and results in a charge-discharge efficiency of 98%. The most prominent peaks for the charging of JUMP-1(Li) are found at 2.1, 1, 0.4, and 0.005 V Li^+^/Li and at 2.3, 1.2, and 0.2 V vs. Li^+^/Li for the discharging (Figure 3D).

The rate capability for the electrodes based on JUMP-1 and JUMP-1(Li) with 1 M LiTFSI in PC is depicted in Figure 3E. For JUMP-1 a discharge capacity of 379 mA∙h∙g^−1^ at 0.1 A∙g^−1^ is found, which is referred to as 100% in this graph. With increasing current densities, a rapid decrease in capacity is observed, which at 10 A∙g^−1^ is only 8% of the initial capacity. On the other hand, JUMP-1(Li) has a much larger discharge capacity of 805 mA∙h∙g^−1^ at 0.1 A∙g^−1^ (set to 100%) and is subject to a less steep decrease in capacity with increasing current, which at a current density of 10 A∙g^−1^ leads to 13% of the initial capacity. From these results it is evident that lithium-ion insertion into the anionic framework prior to electrochemical cycling, as is the case for JUMP-1(Li), leads to an improved insertion process for lithium ions even at elevated current.

Figure 3F shows the stability of the two electrodes based on JUMP-1 and JUMP-1(Li) in 1 M LiTFSI in PC over 1000 charge-discharge cycles carried out with a current density of 1 A∙g^−1^. In the case of JUMP-1, an initial discharge capacity of 152 mA∙h∙g^−1^ is observed, which is in line with the values given in Figure 3B, considering possible minor degradation during the previous measurements. During the first 50 cycles, a loss in specific capacity of 30 mA∙h∙g^−1^ to 120 mA∙h∙g^−1^ is observed for JUMP-1, which, however, stabilizes at this value and leads to a final specific capacity of 124 mA∙h∙g^−1^ after 1000 cycles. A clearly different behavior is observed for the electrode containing JUMP-1(Li). At first, in comparison to JUMP-1, a considerably larger initial discharge capacity of 400 mA∙h∙g^−1^ is measured, which is 90 mA∙h∙g^−1^ lower than the value reported in Figure 3B, again indicating the occurrence of a degradation process of the active material during the measurements performed on the sample prior to this experiment. For JUMP-1(Li), compared to JUMP-1, a faster loss in specific capacity for the electrode of 60 mA∙h∙g^−1^ is observed during the first 25 cycles. Over the following cycling process, a considerably smaller but continuous decrease in capacity was observed, which after 1000 cycles led to a final capacity of 280 mA∙h∙g^−1^, a value that is still twice as high as that of the electrode containing JUMP-1.

**FIGURE 4:**
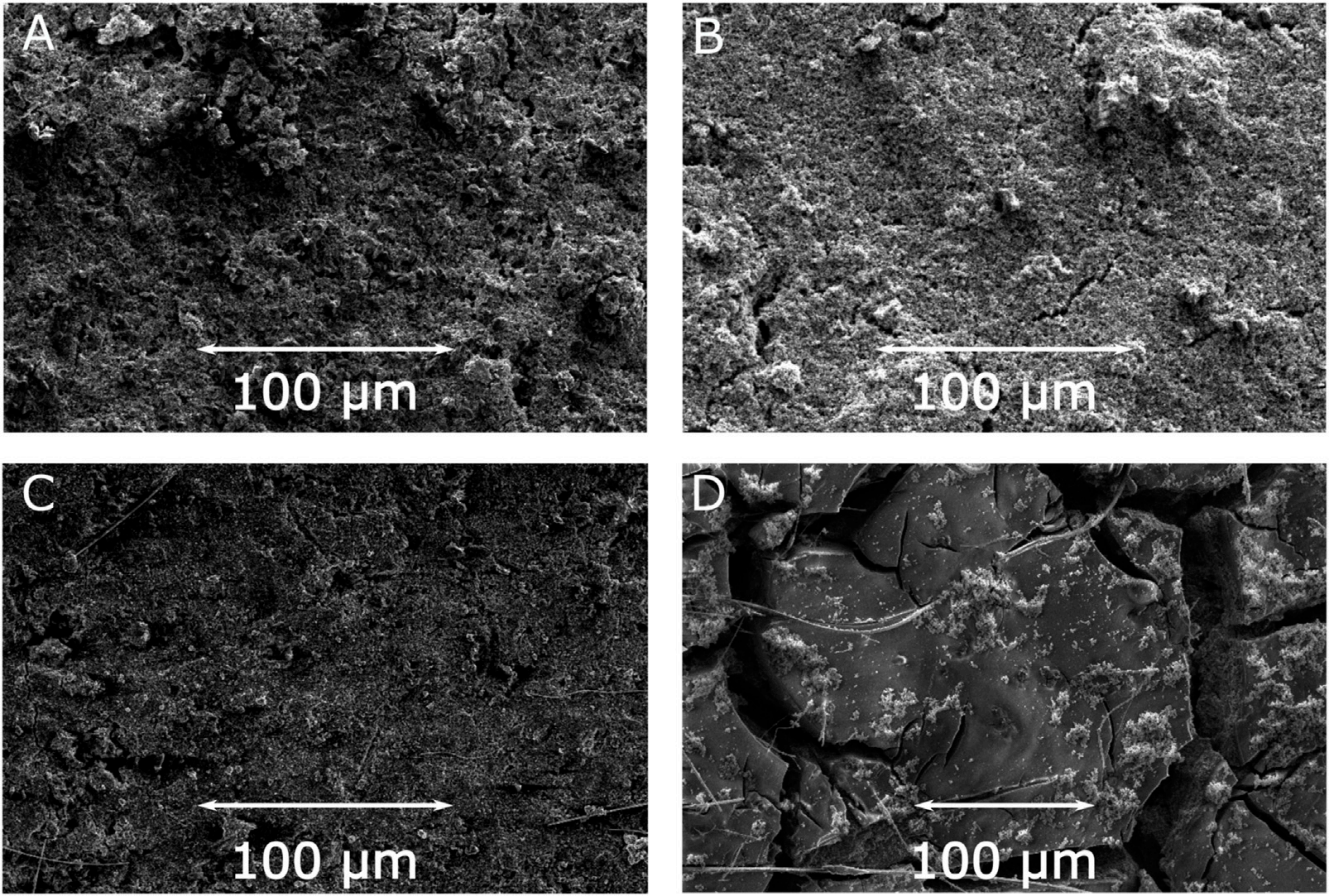
SEM images of electrodes prior to cycling for JUMP-1 **(A)** and JUMP-1(Li) **(B)**, and after cycling for JUMP-1 **(C)** and JUMP-1(Li) **(D)** in 1 M LiTFSI in PC.

For additional insight into the stability of the electrodes with respect to the charge-discharge processes SEM images of the electrodes before and after electrochemical cycling (1000 cycles) were measured. The SEM images of the surface of the electrodes with JUMP-1 and JUMP-1(Li) before and after electrochemical cycling, depicted in Figure 4, reveal certain distinctive features in the morphology of the materials as a result of the repeated charge-discharge processes. An initial comparison of the pristine electrodes of both samples, i.e., JUMP-1 and JUMP-1(Li), prior to cycling shows virtually no differences in their surface structures (Figures 4A,B; Supplementary Figures S4, S5). Moreover, in the case of JUMP-1, no significant changes were visible even after undergoing the large number of charge-discharge processes during the electrochemical cycling (Figure 4C; Supplementary Figure S6). For the electrode containing JUMP-1(Li), however, the SEM images of the electrode surface show distinct changes in its morphology due to the electrochemical cycling (Figure 4D; Supplementary Figure S7). In particular, the surface has become increasingly smooth but with the appearance of an increase in the number and size of cracks in the surface. We attribute these observations to the increased current flow in the electrode with JUMP-1(Li) during the electrochemical cycling, which may have led to the component materials fusing together with the consequence of enlarging the superficial fissures.

To gain better insight into this point we performed EDXS measurements for the electrode containing JUMP-1(Li) before and after the electrochemical cycling. EDXS maps of the pristine electrode reveal the expected initial separation of the MOF crystallites from the rest of the composite, as seen from the localization spots in the cobalt and oxygen maps (Supplementary Figure S8). While the distribution within the carbon map is rather uniform, as one would expect, given that carbon is part of all composite components, the MOF, the conductive carbon, and the polyvinyl fluoride (Supplementary Figure S8). The EDXS measured for the electrode material after electrochemical cycling show that, in contrast to the pristine sample, a virtually uniform distribution within the cobalt and oxygen maps is observed, which are both representative elements of the MOF (Supplementary Figure S9). This supports the hypothesis of a fusion of the component materials based the observed smoothening of the electrode surface, as seen in the SEM image (Figure 4D). As a result, this might explain the rather small nevertheless continuous decrease in capacity observed over the entire cycling range (Figure 3F). On the other hand, SEM images of cross sections measured for electrodes of JUMP-1 and JUMP-1(Li) after 1000 charge-discharge cycles reveal that the morphology of both samples is rather similar with the appearance of subsurface cracks throughout the material (Supplementary Figure S10). Together with the increase of such cracks in number and size, which is evident from the surface SEM images of the material after electrochemical cycling (Figure 4D; Supplementary Figure S7), this may be an additional factor responsible for the observed continuous decrease of the capacity of JUMP-1(Li) as seen in the charge-discharge cycling (Figure 3F).

#### 2.2.2 MOFs as Anodic Materials for SIBs

Sodium-ion batteries (SIBs) emerged as one of the most promising alternatives to LIBs in recent years, and a large number of investigations are nowadays dedicated to these devices. For this reason, we also considered the use of JUMP-1 as electrode material in a sodium-based electrolyte, specifically in 1 M sodium bis(trifluoromethylsulfonyl)imide (NaTFSI) in PC. As in the case of the lithium-based systems reported above, we compared the behavior of JUMP-1 with that of the sodium-exchanged analog JUMP-1(Na).

Again before first measurements, the impedance spectra of both electrodes have been measured. Both electrodes of JUMP-1 and JUMP-1(Na) display similar initial charge transfer resistances (Supplementary Figure S3B).

**FIGURE 5:**
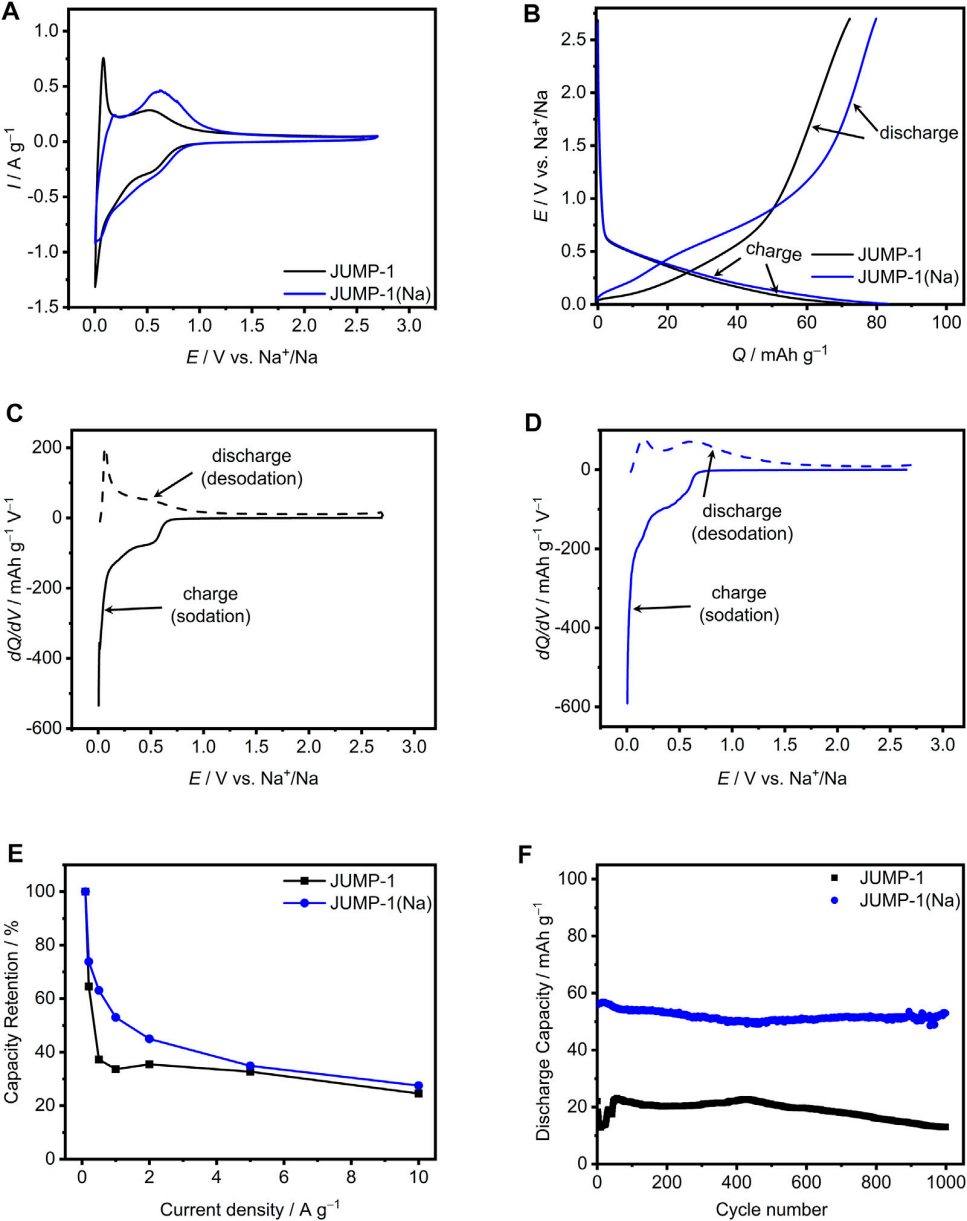
**(A)** CVs at 1 mV∙s^−1^ for JUMP-1 and JUMP-1(Na) in 1 M NaTFSI in PC. **(B)** Galvanostatic charge-discharge profiles at 1 A∙g^−1^ for JUMP-1 and JUMP-1(Na) in 1 M NaTFSI in PC. Differential capacity plots at 1 A∙g^−1^ for JUMP-1 **(C)** and JUMP-1(Na) **(D)** in 1 M NaTFSI in PC. **(E)** Rate capability from 0.1 to 10 A∙g^−1^ of JUMP-1 and JUMP-1(Na) in 1 M NaTFSI in PC. **(F)** Cycling stability at 1 A∙g^−1^ of JUMP-1 and JUMP-1(Na) in 1 M NaTFSI in PC.

Figure 5A shows the CVs of JUMP-1 and JUMP-1(Na) obtained at 1 mV∙s^−1^. The overall measured current density of both materials is comparable, which is different for the case when lithium ions were used as charge carriers. In the range from 1.1 to 2.7 V vs. Na^+^/Na no redox peaks are present for either of the electrodes, and at lower potential sloping profiles were observed for both cases. Nevertheless, JUMP-1 shows a very distinct peak at 0.1 V vs. Na^+^/Na during the sodium extraction process, which was not obtained with JUMP-1(Na). In contrast, JUMP-1(Na) shows a very pronounced peak at 0.63 V vs. Na^+^/Na, which is less distinct for JUMP-1. The galvanostatic charge-discharge profiles depicted in Figure 5B show a similar cycling profile and comparable capacities of 73 mA∙h∙g^−1^ and 83 mA∙h∙g^−1^ for JUMP-1 and JUMP-1(Na), respectively. Considering these results, also in the case of the sodium ion systems the use of the ion-exchanged MOF JUMP-1(Na) still provides a higher specific capacity compared to original JUMP-1, with dimethylammonium cations in the pores of the MOF. However, in this case this effect is considerably less pronounced than in the case of lithium.

The faradaic storage behavior of JUMP-1 and JUMP-1(Na) in 1 M NaTFSI in PC is depicted in Figures 5C,D, which is seen to differ significantly from that observed for the corresponding electrodes of the lithium-ion based systems (Figures 3C,D). For JUMP-1 basically no storage process is present in the potential range of 1–2.7 V vs. Na^+^/Na (Figure 5C), whereas below 1 V vs. Na^+^/Na there are two peaks for faradaic storage in the sodium insertion process at 0.5 and 0.005 V vs. Na^+^/Na and two corresponding peaks in the sodium release, which are very pronounced at 0.07 and 0.5 V vs. Na^+^/Na. The relevant areas of the sodium ion insertion and release result to 73 and 72.5 mA∙h∙g^−1^, respectively, which yields a cycling efficiency of 99%.

For JUMP-1 (Na) a basically similar behavior is observed, as depicted in Figure 5D, with no faradaic storage provided by the sample at potentials higher than 1.3 V vs. Na^+^/Na. Nonetheless, there is a small but significant difference due to the missing pronounced discharge peak at very low potential as compared to non-ion exchanged analog JUMP-1 (at 0.07 V vs. Na^+^/Na). The peaks observed for the charging of JUMP-1 (Na) are at 0.5 and 0.005 V vs. Na^+^/Na with a charge area of 83 mA∙h∙g^−1^, while for the discharge process the peaks are at 0.2 and 0.6 V vs. Na^+^/Na with a total charge of 80 mA∙h∙g^−1^, which concludes to a cycling efficiency of 96%.

Figure 5E depicts the rate capability for the electrodes based on JUMP-1 and JUMP-1(Na) from 0.1 to 10 A∙g^−1^ in 1 M NaTFSI in PC as electrolyte. The discharge capacity for JUMP-1 at 0.1 A∙g^−1^ is found to be 110 mA∙h∙g^−1^ at 0.1 A∙g^−1^, which is referred to as 100% in this graph. As shown in Figure 3E, the capacity of the electrode decreases significantly up to a current density of 0.5 A∙g^−1^ and, after stabilization at a weak minimum, it falls again with a continuous decrease until it reaches 25% of the initial value. In contrast, a less steep initial decrease in capacity is visible for JUMP-1(Na), with a behavior similar to that of the Li analogs (see Figure 3E), but with lower absolute discharge capacity of 149 mA∙h∙g^−1^ at 0.1 A∙g^−1^. At a current density of 10 A∙g^−1^, 41% of the initial capacity are still accessible. Thus, also in the case of the sodium-ion based electrolyte systems, the cation exchange in the MOF is beneficial for the performance of the corresponding electrode with JUMP-1(Na) during charge-discharge cycling.

The cycling stability of the electrodes containing JUMP-1 and JUMP-1(Na) in 1 M NaTFSI in PC over 1000 charge-discharge cycles carried out with a current density of 1 A∙g^−1^ is presented in Figure 5F. In the case of JUMP-1, a discharge capacity of 22 mA∙h∙g^−1^ is found, which is significantly lower than that observed when the same electrode was cycled in lithium-based electrolyte (152 mA∙h∙g^−1^, see Figure 3F). It is interesting to note here that this observed initial capacity of 22 mA∙h∙g^−1^ is only about 30% of the capacity determined from galvanostatic charge-discharge profiles (Figure 5B). This clearly indicates that already the electrochemical measurement carried out prior to the cycling stability test caused a dramatic loss in performance of the electrode. Moreover, the capacity even drops to a value of 13 mA∙h∙g^−1^ within the first few cycles, which then recovers to a value of 23 mA∙h∙g^−1^ during the first 50 cycles. The capacity remains above 20 mA∙h∙g^−1^ up to about 550 cycles and decreases after the full 1000 cycles to a final value of 13 mA∙h∙g^−1^. This behavior is most likely caused by the larger size of the sodium ions compared to that of the lithium ions, leading to a more stressful charge-discharge process for the host structure. In this context, is worth noting that the PXRD pattern for the sodium-ion exchanged JUMP-1(Na) also indicated slight structural changes in the crystalline material (Supplementary Figure S2).

A clearly different behavior is obtained for the electrode containing the JUMP-1(Na). Although, as in the case of JUMP-1, a reduced discharge capacity of 56 mA∙h∙g^−1^ was measured at the beginning of the experiment compared to the that obtained from the galvanostatic charge-discharge profiles (83 mA∙h∙g^−1^, see Figure 5B), only minor degradation and loss in capacity was observed during the full cycling process with a final capacity of 53 mA∙h∙g^−1^. Albeit the capacity of the electrodes containing JUMP-1 and JUMP-1(Na) is overall lower than that observed for the lithium-based systems, the comparison of their behavior still proves a stabilization of the host structure through the pre-alkalation treatment that replaces the dimethylammonium by the sodium ions.

**FIGURE 6:**
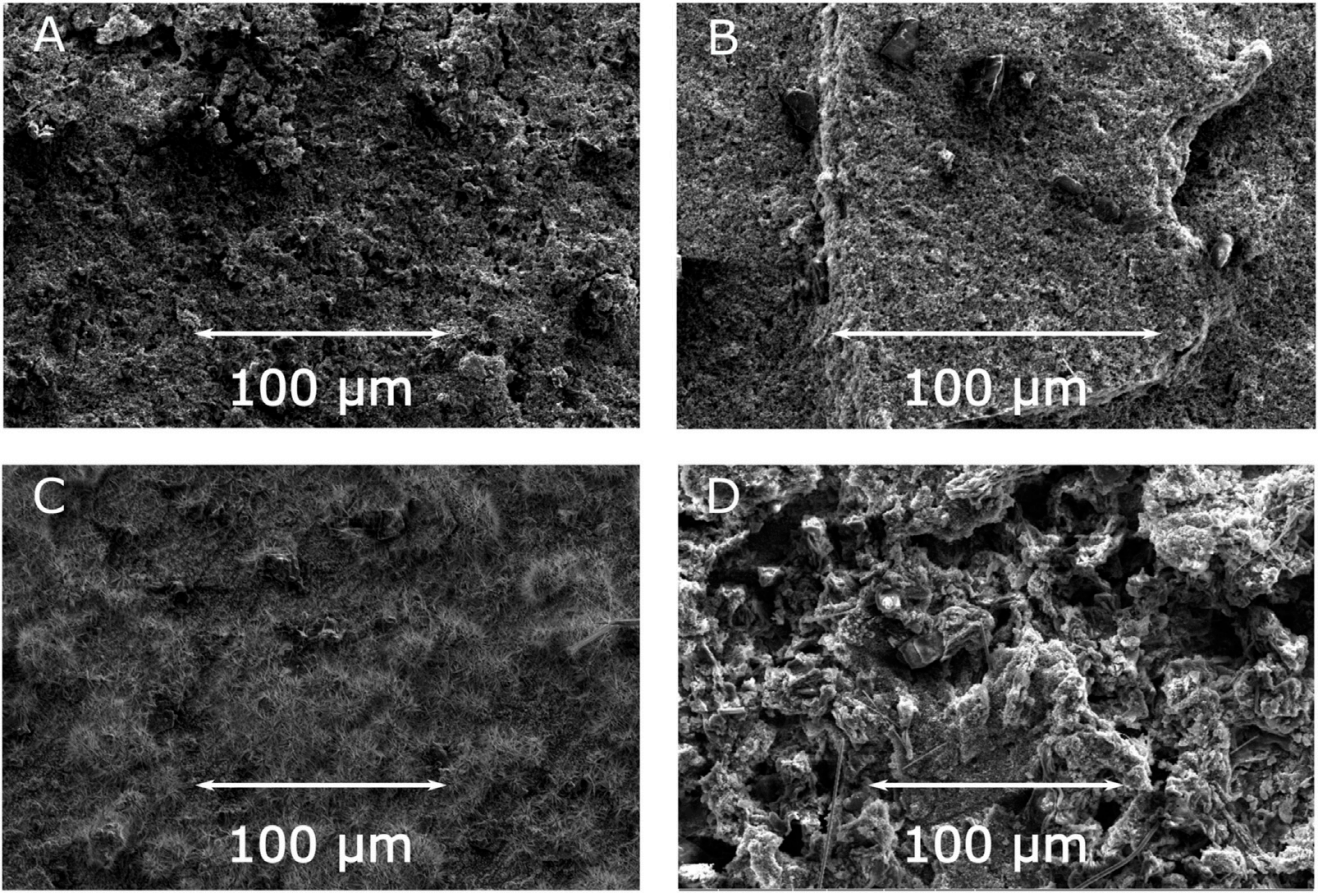
SEM images of electrodes prior to cycling for JUMP-1 **(A)** and JUMP-1(Na) **(B)**, and after cycling for JUMP-1 **(C)** and JUMP-1(Na) **(D)** in 1 M NaTFSI in PC.

In order to shed some light on the morphological aspects of the above results, SEM images of the relevant electrodes before and after electrochemical cycling (1000 cycles) were measured. Figure 6 depicts the surface SEM images of the electrodes with JUMP-1 and JUMP-1(Na) as-prepared and after electrochemical testing. As expected, the comparison of the as-prepared electrodes containing JUMP-1 and JUMP-1(Na) show no differences in their basic surface structures (Figures 6A,B; Supplementary Figures S4, S11). In contrast to this, however, a distinct change in the morphology of the electrode material is observed in both cases after the electrochemical cycling processes (Figures 6C,D; Supplementary Figures S12, S13), which clearly indicates a deterioration of the composite materials. This is in accordance with the electrochemical measurements, which show a drastic loss in capacity already during the first charge-discharge cycles.

In the case of JUMP-1, the severe deterioration led to an overall loss of integrity of the electrode material, which can be seen in the top view SEM images from the fact that, even though remains of the glass fiber separator are visible, the coverage of the basal copper foil by the composite electrode material is partially lost (Figure 6C; Supplementary Figure S12). This is confirmed by SEM image of the cross section measured for the electrode with JUMP-1 after charge-discharge cycling (Supplementary Figure S14A), which shows the presence of the almost bare supporting copper foil for the selected area. The electrode with the sodium-ion exchanged JUMP-1(Na), on the other hand, did not suffer as much from the extended charge-discharge cycling, which can be attributed to increased pore accessibility due to the cation exchange prior to the electrochemical treatment. This observation is confirmed by the cross-sectional SEM image of the electrode with JUMP-1(Na) after cycling (Supplementary Figure S14B), that shows a considerably lower degree of deterioration than that observed for the non-ion exchanged JUMP-1 case. Nevertheless, in contrast to the cases of the lithium system, a significantly increased roughness in its surface morphology is observed, when compared with the electrode prior to the electrochemical cycling (Figures 6B,D).

## 3 Conclusion

The anionic MOF JUMP-1, with a pillared-layer architecture, was investigated as anode material for lithium and sodium ion batteries. A particular focus was placed on the nature of the cation balancing the charge of the anionic MOF. In addition to JUMP-1 with the dimethylammonium cation, the ion-exchanged analogs JUMP-1(Li) and JUMP-1(Na) were also investigated, with lithium and sodium counterions balancing the anionic porous framework. A comparison of these electrode materials generally shows that the absence of dimethylammonium cation in the pores of the anionic framework leads to a significantly improved electrochemical performance and mechanical stability of the relevant electrode materials. In other words, pre-loading the anionic MOF with the appropriate cations, that are used as charge carriers in the relevant system, is beneficial for the electrode performance. However, there is a major difference in the overall performance and stability of the anionic framework of JUMP-1, depending on whether lithium or sodium is used in the electrodes. Although neither the choice of additive composite materials nor the electrolyte have been optimized for the system, JUMP-1(Li) shows a remarkable capacity and good stability over prolonged charge-discharge. On the other hand, the results for the sodium systems clearly show, that charge-discharge processes of the anionic framework with sodium ions are more stressful for the electrode composite material leading to a loss of the mechanical integrity.

## 4 Experimental Section

### 4.1 Methods

Simultaneous TG/DTA analyses were performed under static air atmosphere using a Netzsch STA Luxx PC analyzer up to 1000 °C. The FT-IR spectra were measured on a VERTEX 70 IR spectrometer by Bruker Optics using the Specac Diamond ATR optional accessory. The elemental analyses were done on a VARIO EL III analyzer. Solvothermal reactions were carried out in a 23 ml Teflon-lined acid digestion vessel from Parr Instruments, utilizing a programmable oven by Binder. The argon physisorption isotherms were measured on an Autosorb-IQ instrument from Anton Paar. Powder X-ray diffraction (PXRD) measurements were performed on a Stoe Powder Diffractometer with a Mythen 1K detector at room temperature. Measurements were done using capillary tubes while the Debye Scherrer Scan Mode was applied with a 2θ scan type. The X-ray tube was a Cu-long fine focus tube. The powdered samples were placed in a 0.5 mm glass capillary and then measured. The measurement was carried out between 2 and 50° with steps of 2.1° per 20 s. EDXS measurements of the composite electrodes were measured using a FEI dual beam FIB with a high-resolution electron beam. The beam energy and current were set to 15 keV and 0.69 nA, respectively.

### 4.2 Materials

4-fluorobenzonitrile (Alfa Aesar), 4-aminobenzonitrile (Alfar Aesar), and cobalt (II) chloride hexahydrate (Aldrich) were obtained commercially and used without further purification. All other chemicals were of AR grade.

### 4.3 MOF Syntheses

#### 4.3.1 ((CH_3_)_2_NH_2_)_2_[Co_3_(ntb)_2_bdc]∙5H_2_O (JUMP-1)

JUMP-1 was synthesized as previously reported (Akintola et al., 2017b) and then washed repeatedly with DMF to remove any unreacted ligand or metal salt. The violet crystals were then soaked in ethanol for 7 days, during which the solvent was replaced daily and then dried using supercritical CO_2_ according to the autoclave method employed for larger amounts of bulk material on a gram scale. Elemental analysis calcd for JUMP-1 C_54_H_54_Co_3_N_4_O_21_ (*M* = 1272 g∙mol^−1^): C, 51.00; H, 4.08; N, 4.20%. Found: C, 51.19; H, 4.28; N, 4.41%. Selected IR data (ATR, cm^−1^): 1590vs, 1538s, 1505s, 1379vs, 1313s, 1273s, 1173m, 1104w, 842w, 779vs, 701w, 675w, 517s.

#### 4.3.2 Li_2_[Co_3_(ntb)_2_bdc]∙8H_2_O (JUMP-1(Li))

JUMP-1(Li) was obtained by immersing the as-synthesized JUMP-1 in a saturated solution of LiNO_3_ in DMF for 7 days followed by repeated washing with the solvent. The Li-exchanged material was then soaked in ethanol for another 7 days and then dried using supercritical CO_2_ according to the autoclave method employed for larger amounts of bulk material on a gram scale. Elemental analysis calcd for JUMP-1(Li) C_50_H_44_Co_3_N_2_O_24_Li_2_ (*M* = 1248 g∙mol^−1^): C, 48.14; H, 3.55; N, 2.24%. Found: C, 48.02; H, 3.60; N, 2.04%. Selected IR data (ATR, cm^−1^): 3425br, 1588s, 1504m, 1378vs, 1313s, 1275s, 1174m, 1147w, 1088w, 1045w, 847w, 780s, 676m, 518s.

#### 4.3.3 Na_2_[Co_3_(ntb)_2_bdc]∙8H_2_O (JUMP-1(Na))

JUMP-1(Na) was synthesized according to the procedure described for JUMP-1(Li), but using NaNO_3_ for ion exchange. It was subsequently treated in the same manner as JUMP-1(Li) and then dried using supercritical CO_2_ according to the autoclave method employed for larger amounts of bulk material on a gram scale. Elemental analysis calcd for JUMP-1(Na) C_50_H_44_Co_3_N_2_O_24_Na_2_ (*M* = 1280 g∙mol^−1^): C, 46.92; H, 3.47; N, 2.19%. Found: C, 47.03; H, 3.46; N, 2.06%. Selected IR data (ATR, cm^−1^): 3397br, 1588s, 1551s, 1505m, 1378vs, 1314s, 1271s, 1174m, 1147w, 1045w, 826w, 780s, 747s, 676m, 511s.

### 4.4 Supercritical Carbon Dioxide Treatment

#### 4.4.1 Autoclave Method for Bulk Material in Gram Scale

About 1 g of the material was immersed in ethanol (100 ml) for 7 days during which the solvent was refreshed daily. After immersion, the supernatant ethanol was removed and the ethanol-MOF slurry, after the supernatant solvent left only a thin film of ethanol (to ensure sample did not dry out during transfer), was transferred into an autoclave and sealed. Liquid CO_2_ was then introduced into the autoclave (100 ml at a pressure of 60 bar) and allowed to stand 30 min after which the CO_2_ was then slowly released from the autoclave over a period of 20 min to remove any possible non-occluded ethanol from the materials. Subsequently liquid CO_2_ was reintroduced into the reactor and this time allowed to stand for 24 h. After this period, the temperature of the autoclave was raised to 40 °C to bring the CO_2_ to supercritical conditions and maintained for 1 hour. The gas was then slowly released over 30 min with the temperature maintained at 40 °C to prevent any cooling that might result from expansion of the gas during evaporation.

#### 4.4.2 MOF Pretreatment for Sorption Measurements

Prior to sorption measurements all samples were dried using supercritical CO_2_. The drying procedure was performed using a K850 Critical Point Dryer provided by Quorum Technologies according to the following procedure.

About 40–50 mg of the material was immersed in ethanol (10 ml) for 7 days during which the solvent was refreshed daily. The supernatant was decanted off and the samples carefully transferred into the small porous pots and then into the sample holder. The drying chamber was at this point precooled to 5 °C, after which the sample was quickly transferred into the chamber and then hurriedly but carefully sealed tight. This was followed by filling up the chamber with liquid CO_2_ and then stirring while holding for 30 min. After this, the stirring was stopped, and the liquid CO_2_ was allowed to slowly drain off. The chamber was re-cooled down to 5 °C and then refilled with liquid CO_2_ and this time allowed to stand for 24 h while stirring. At the end of the 24 h period, the stirrer was turned off and the chamber was once again slowly emptied and allowed to briefly stand empty. For a third time, the chamber was cooled down to 5 °C and then filled while stirring for another 1 hour followed by slow release. In a final run, after cooling the chamber down and then filling with liquid CO_2_, the heater was started and after 35 min, the CO_2_ was brought to supercritical conditions and maintained for a further 90 min. The gas was then slowly released while keeping the heater on to prevent freezing.

### 4.5 Argon Sorption Measurements

The isotherms of all pre-treated and dried products were measured immediately after outgassing the samples for 30 min at room temperature using argon at 87 K. Pore size distribution curves were calculated by fitting the experimental data using a Non-local density functional theory (NLDFT) kernel based on adsorption models for argon on zeolites/carbon at 87 K with cylindrical pores, which was provided by QUANTACHROME Instruments (QUANTACHROME). The Brunauer–Emmett–Teller (BET) surface areas for all materials were determined from the adsorption data over different relative pressure ranges all between 0.007 and 0.35 while ensuring compliance with the consistency criteria (see Supplementary Tables S1, S2) (Rouquerol et al., 2007).

### 4.6 Electrode Preparation

For electrode preparation, all MOF samples were dried using supercritical CO_2_ according to the autoclave method employed for larger amounts of bulk material on a gram scale. This was in order to have clean dry surfaces in the materials while still maintaining their porosities.

The electrodes used for the electrochemical measurements are based a Swagelok-type cell design in a 3-electrode-configuration where the MOF composite electrode was used as working electrode (WE), elemental lithium or sodium was used as counter electrode (CE) and as reference (Ref). WE, CE and Ref have been separated by a glass fiber separator, which was drenched with 150 µl of electrolyte.

The WEs were prepared by mixing the appropriate activated MOF with carbon black as conductive additive and polyvinyl fluoride as binder in a ratio of 65:30:5 using N-methyl-2-pyrrolidone to obtain a homogenous slurry. This was then cast onto copper foils and dried with a wet film thickness of 250 µm. After drying, the average mass loading resulted to 0.84 g∙cm^−2^ for JUMP-1, 0.93 g∙cm^−2^ for JUMP-1(Li) and 0.47 g∙cm^−2^ for JUMP-1(Na). Electrolytes were prepared by either dissolving 1 M lithium bis(trifluoromethysunflonyl)imide (LiTFSI) or sodium TFSI (NaTFSI) in propylene carbonate (PC). All solvents, salts and electrolytes were prepared and stored in a glovebox (Labmaster, MBRAUN GmbH) with an argon atmosphere with a water and oxygen content below 0.1 ppm.

### 4.7 Electrode Characterization

Before starting the measurements, 3 h of open circuit voltage (OCV) were recorded to set the systems into an equilibrium after assembly. At first Cyclic voltammogram tests were carried out between 0.005 and 3 V vs. Li^+^/Li or 0.005 and 2.7 V vs. Na^+^/Na using a scan rate ranging from 0.1—100 mV/s (for a total of 75 cycles). Subsequent 100 galvanostatic charge/discharge cycles were carried out between 0.005 and 3 V vs. Li^+^/Li or 0.005 and 2.7 V vs. Na^+^/Na with a current density ranging from 1 A∙g^−1^.

Afterwards galvanostatic charge/discharge tests were carried out between 0.005 and 3 V vs. Li^+^/Li or 0.005 and 2.7 V vs. Na^+^/Na with current densities ranging from 0.1 to 10 A∙g^−1^ and 100 cycles for each used current density (800 cycles in total).

At last, the cycling stability has been measured for 1000 cycles at a current density of 1 A∙g^−1^, which in total sums up to 1975 cycles done with each electrode. The stability measurements show the cycles 976–1975.

Between different measurements the impedance of the electrodes have been investigated using an amplitude of 5 mV and frequencies between 500 kHz and 10 mHz.

All electrochemical measurements were carried with a VMP multichannel potentiostatic-galvanostatic workstation (Biologic Science Instruments, VMP 3) or an Arbin potentiostatic-galvanostatic workstation (Arbin instruments, LBT21084) at room temperature. Current densities and specific capacities were calculated based on the mass of the MOF active material in the WE.

Before and after all electrochemical measurements, SEM images and EDXS elemental maps of the electrodes were acquired along their cross-section and in top-view geometry to investigate their morphologies and distribution of elements. The cross-sections were obtained by simply cutting the electrodes in half. A small region (25–60 µm) was then selected and smoothened by sputtering using a 30 keV Ga ion beam from the FIB. Acquisition of the EDXS elemental maps was then performed at 15 keV.

## Data Availability

The raw data supporting the conclusion of this article will be made available by the authors, without undue reservation.
