# Supplementary material for: Enhancing Capacity and Stability of Anionic MOFs as Electrode Material by Cation Exchange
Source: Front Chem. 2022 Mar 4;10:836325. doi: 10.3389/fchem.2022.836325 (PMC8942763; doi:10.3389/fchem.2022.836325)
Supplement: Supplementary file 1 [file DataSheet1.docx]

Supplementary Material

# Differential thermogravimetric measurements on all three samples


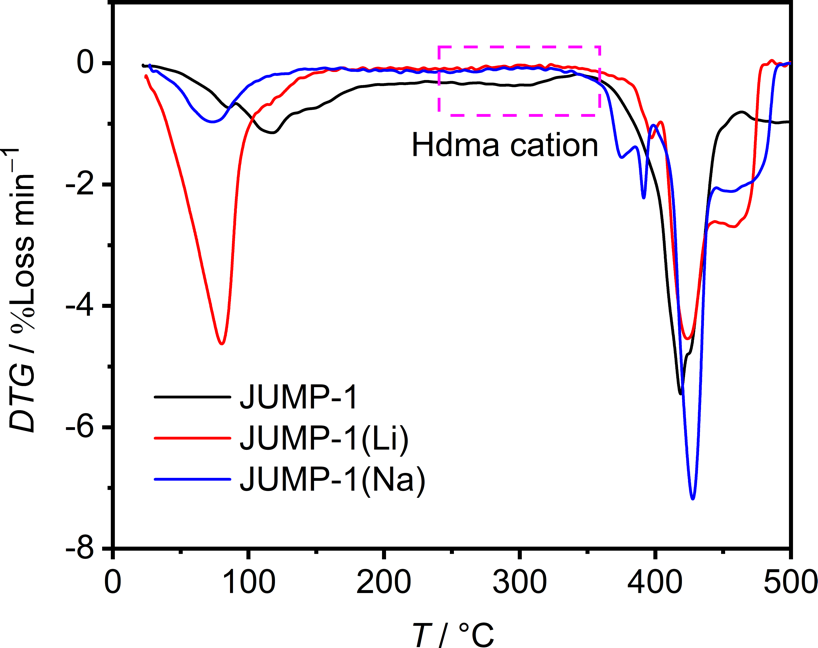


**Figure S1**. DTG data of the as-synthesized JUMP-1 and the cation-exchanged samples JUMP-1(Li) and JUMP-1(Na). The temperature range with indicative features attributed to the presence of dimethylammonium cations (Hdma) is highlighted by the magenta box with broken lines.

# Powder X-ray diffraction (PXRD)


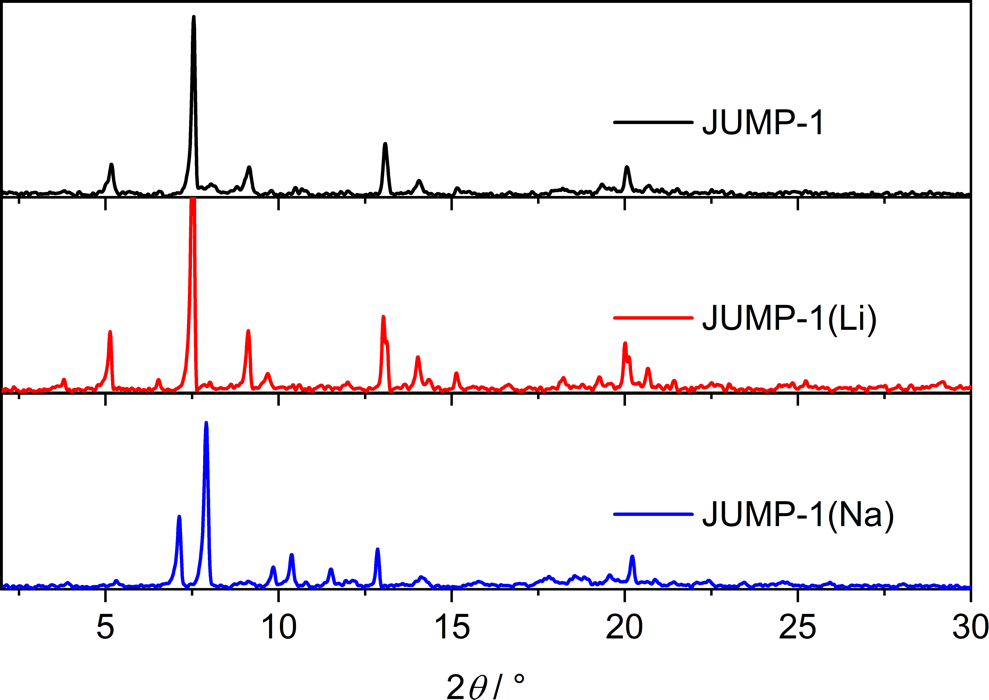


**Figure S2**. PXRD pattern of the as-synthesized JUMP-1 and the cation-exchanged samples JUMP-1(Li) and JUMP-1(Na).

# Sorption data summary

**Table S1**. Summary of sorption data derived from argon sorption isotherms of the as-synthesized JUMP-1 and the cation-exchanged samples JUMP-1(Li) and JUMP-1(Na).

|  | JUMP-1 | JUMP-1(Li) | JUMP-1(Na) |
| --- | --- | --- | --- |
| *a*BET (m^2^∙g^−1^) | 100 | 420 | 180 |
| Total pore volume (cm^3^∙g^−1^) | 0.10 | 0.26 | 0.20 |
| Modal pore width (nm) | 3.9 | 1.1 | 1.3 |

**Table S2**. Consistency criteria for calculating BET surface areas for as-synthesized JUMP-1 and the cation-exchanged samples JUMP-1(Li) and JUMP-1(Na).

|  | JUMP-1 | JUMP-1(Li) | JUMP-1(Na) |
| --- | --- | --- | --- |
| $\frac{P}{P_{0}}$ range | 0.20–0.35 | 0.007–0.100 | 0.13–0.2 |
| *C* | 3.8 | 1723.5 | 53.9 |
| *V*_m_ (cm^3^∙g^−1^) | 25.9 | 110.5 | 48.1 |
| $\frac{1}{\surd C+1}$ | 0.34 | 0.024 | 0.123 |
| $\frac{P}{P_{0}}$ (*V*_m_) (cm^3^∙g^−1^) | 0.34 | 0.025 | 0.120 |
| *a*BET (m^2^∙g^−1^) | 100 | 420 | 180 |
| *R* | 0.999 | 0.9999 | 0.9999 |

# Impedance spectra of pristine electrodes run in 1M LiTFSI and NaTFSI in PC


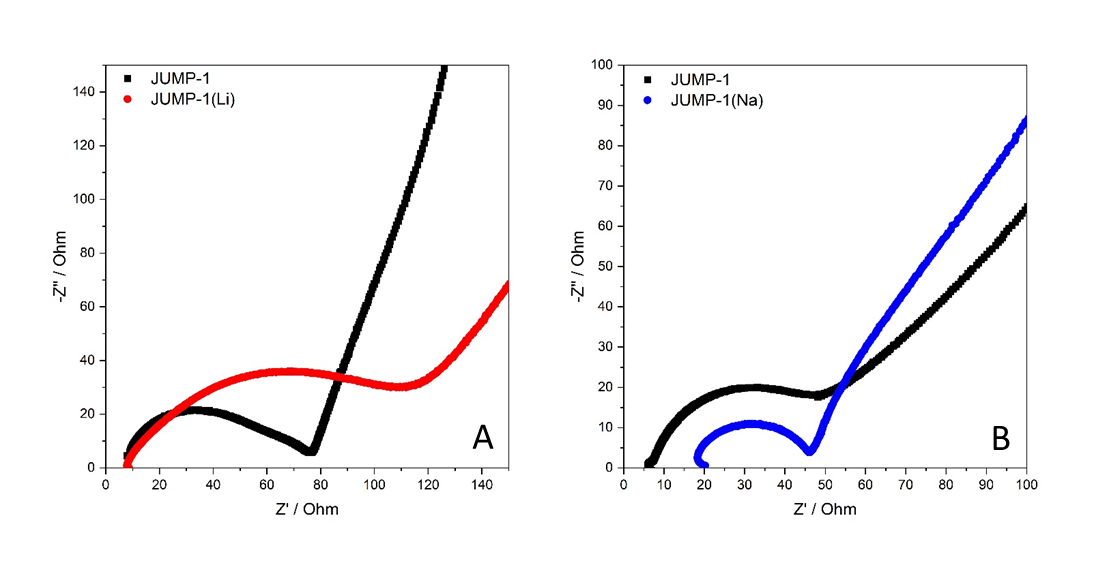


**Figure S3**. Impedance spectra of JUMP-1 and JUMP-1(Li) in 1 M LiTFSI in PC (**A**) and JUMP-1 and JUMP-1(Na) in 1 M NaTFSI in PC (**B**) before electrochemical measurements.

# SEM images of electrodes of the lithium system


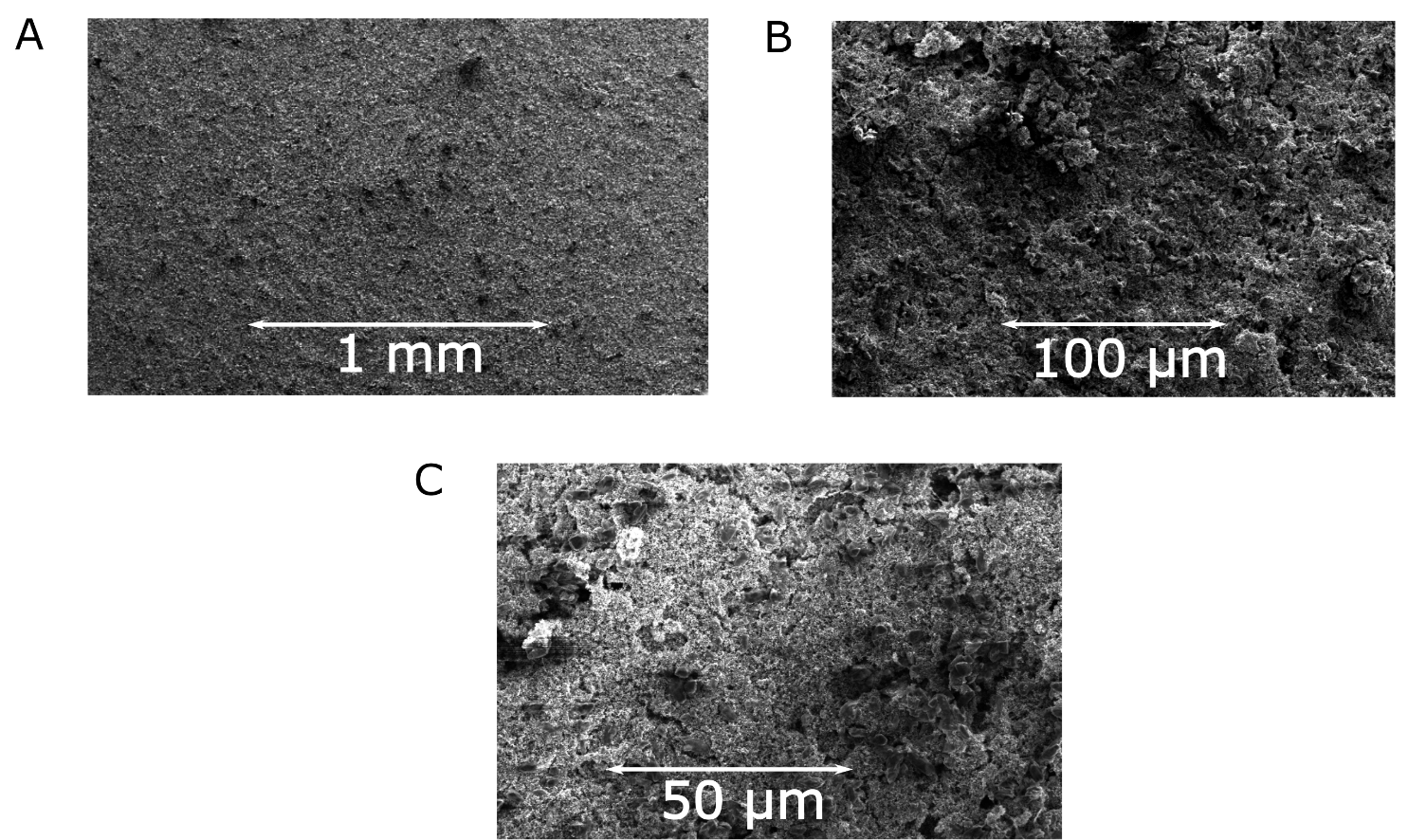


**Figure S4**. SEM images of the surface of the electrode with JUMP-1 before electrochemical measurements shown with different magnifications.


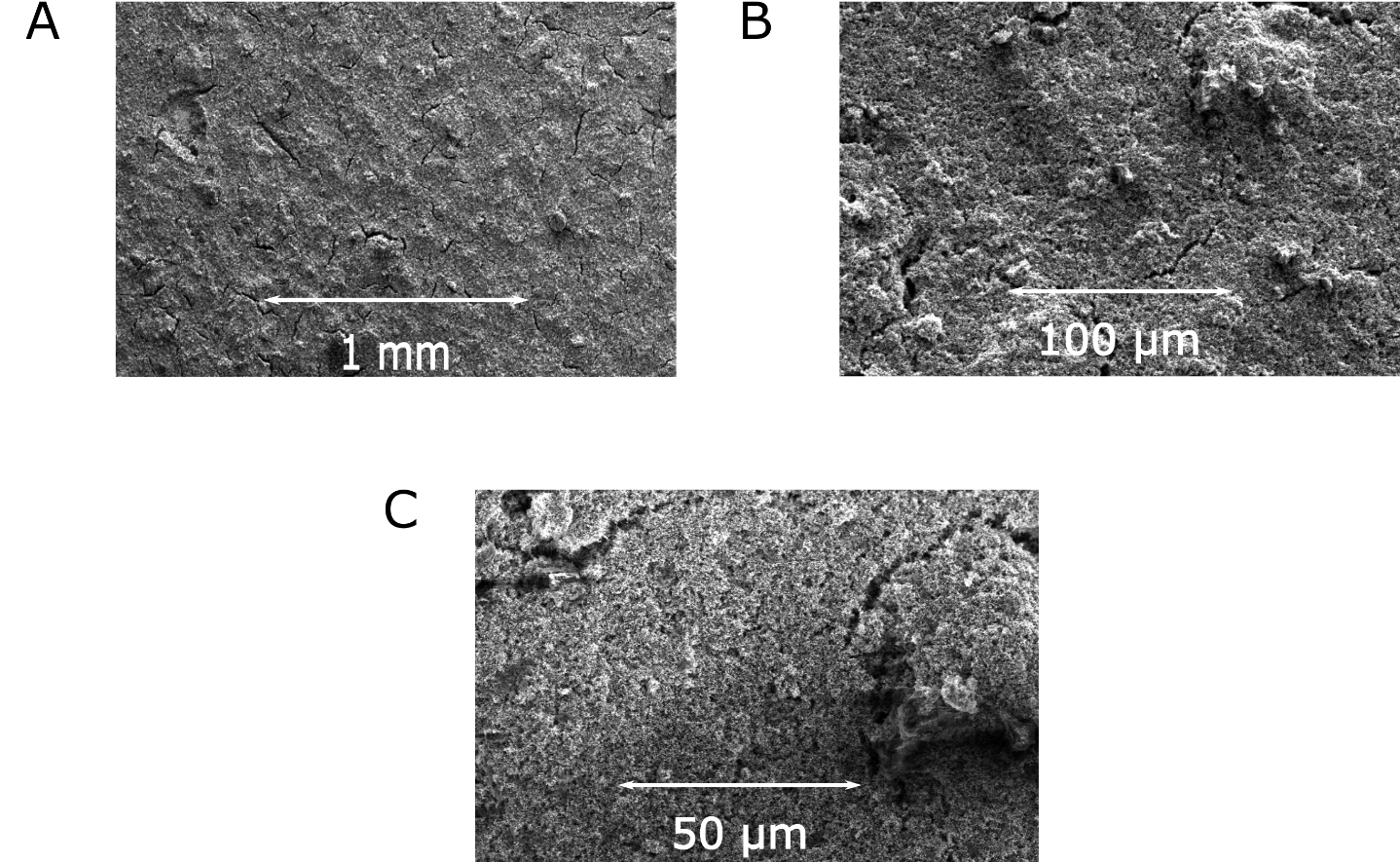


**Figure S5**. SEM images of the surface of the electrode with JUMP-1(Li) before electrochemical measurements shown with different magnifications.


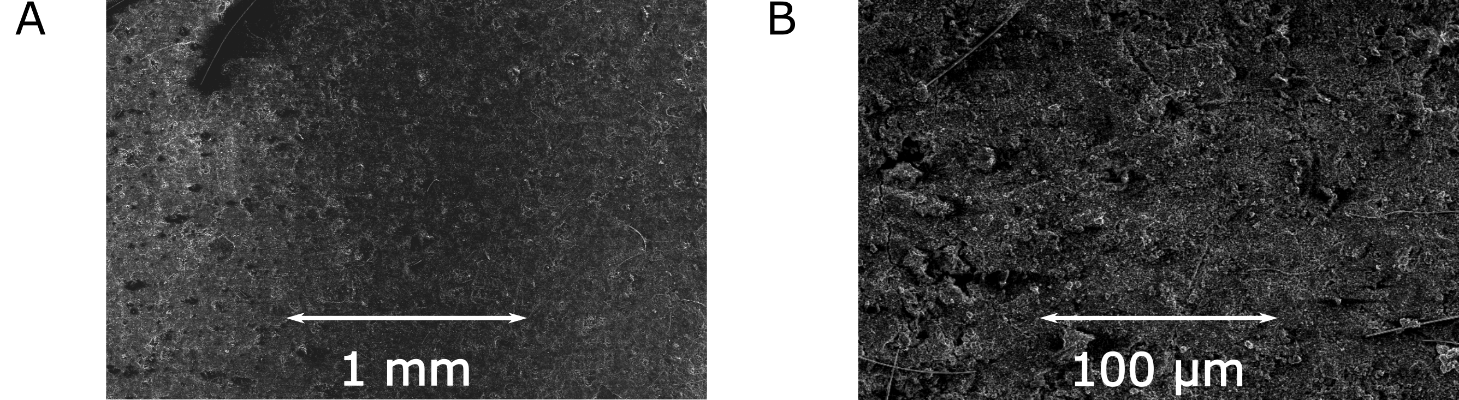


**Figure S6**. SEM images of the surface of the electrode with JUMP-1 after 1000 charge-discharge cycles using 1 M LiTFSI in PC shown with different magnifications.


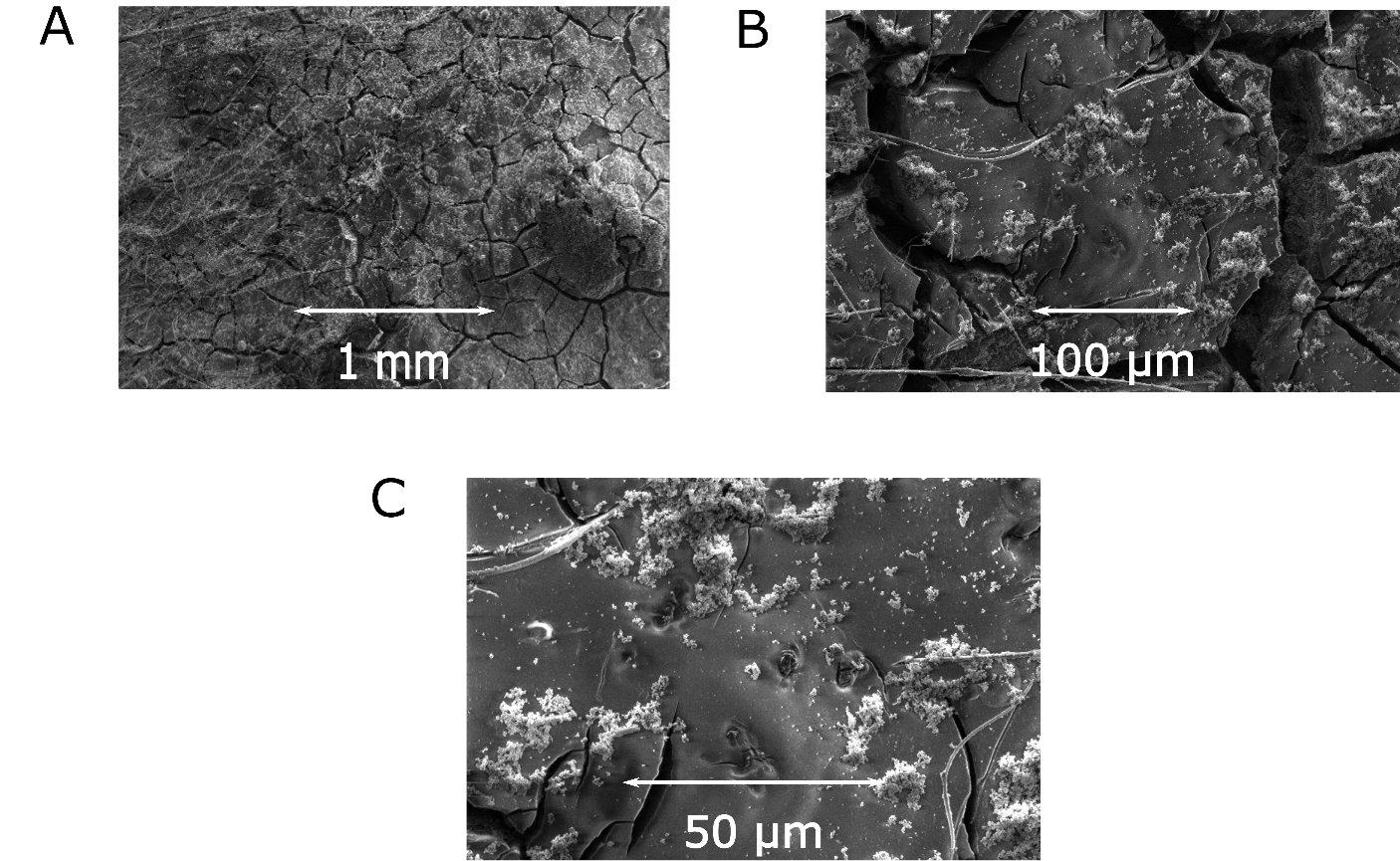


**Figure S7**. SEM images of the surface of the electrode with JUMP-1(Li) after 1000 charge-discharge cycles using 1 M LiTFSI in PC shown with different magnifications.

#
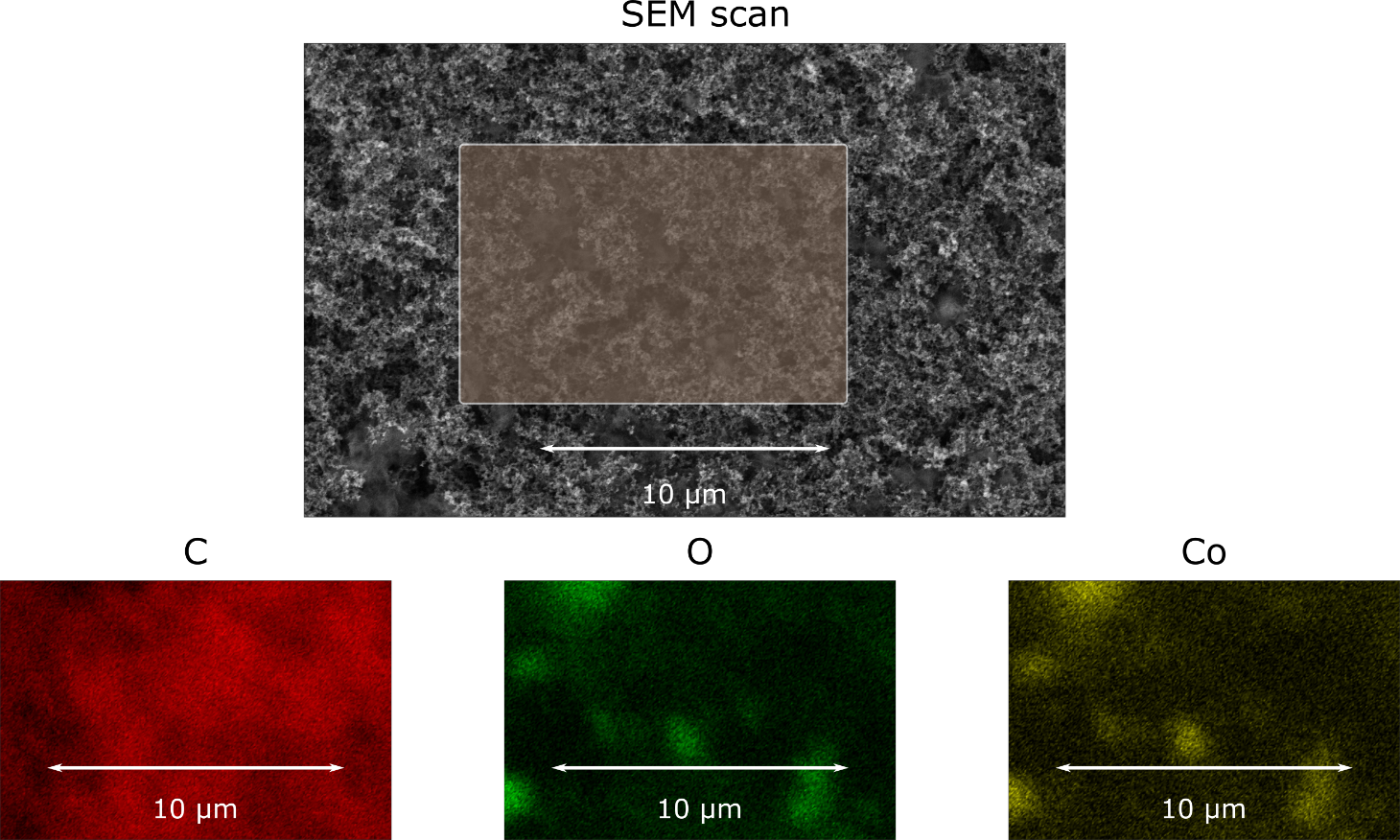
Comparative EDXS elemental maps of JUMP-1(Li) prior to and after charge-discharge cycling

**Figure S8**. EDXS elemental maps for the top view of the surface of the electrode with JUMP-1(Li) prior to its charge-discharge cycling using 1 M LiTFSI in PC.


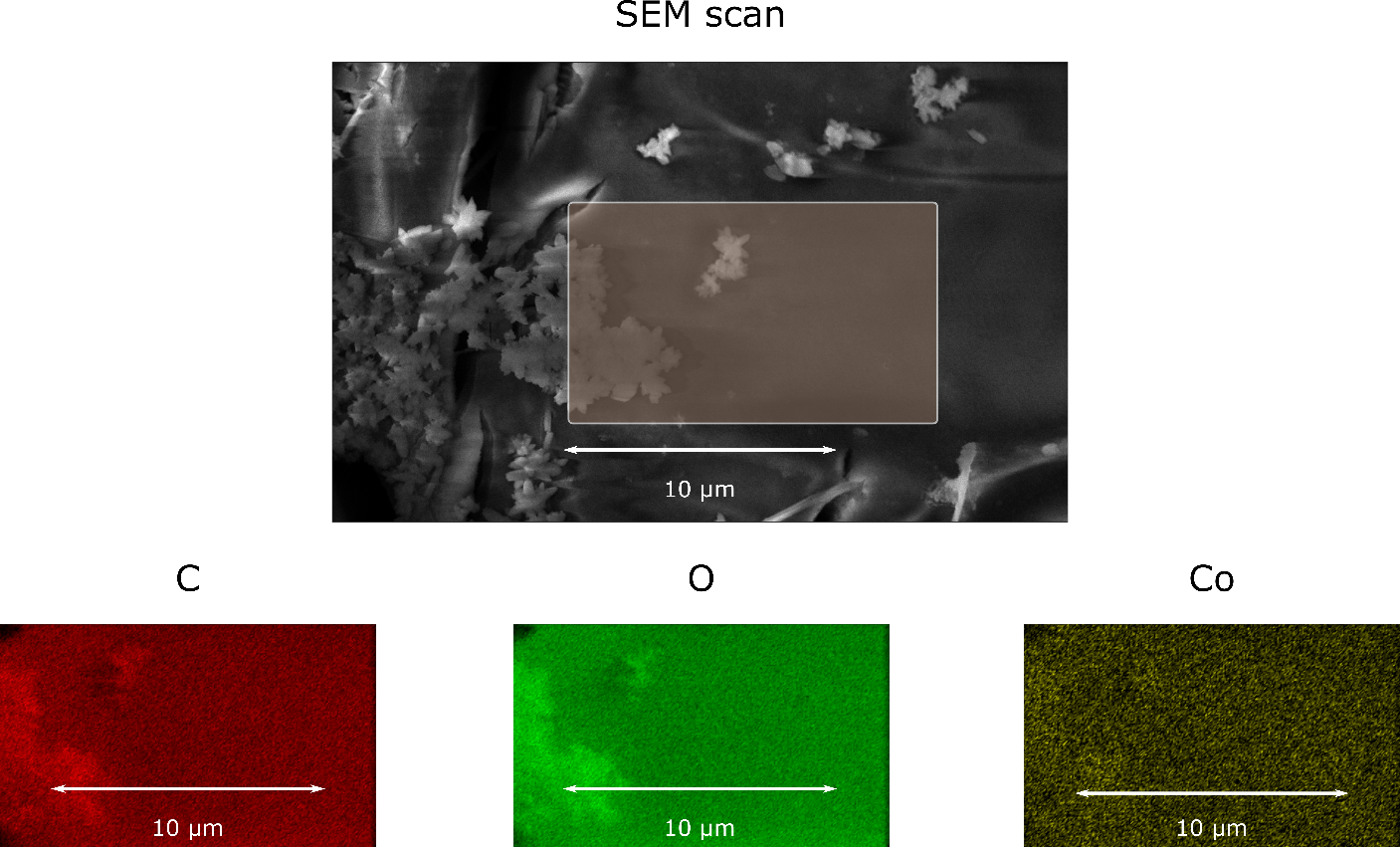


**Figure S9**. EDXS elemental maps for the top view of the surface of the electrode with JUMP-1(Li) after 1000 charge-discharge cycles using 1 M LiTFSI in PC.

# Cross-sectional SEM images of electrodes cycled in LiTFSI


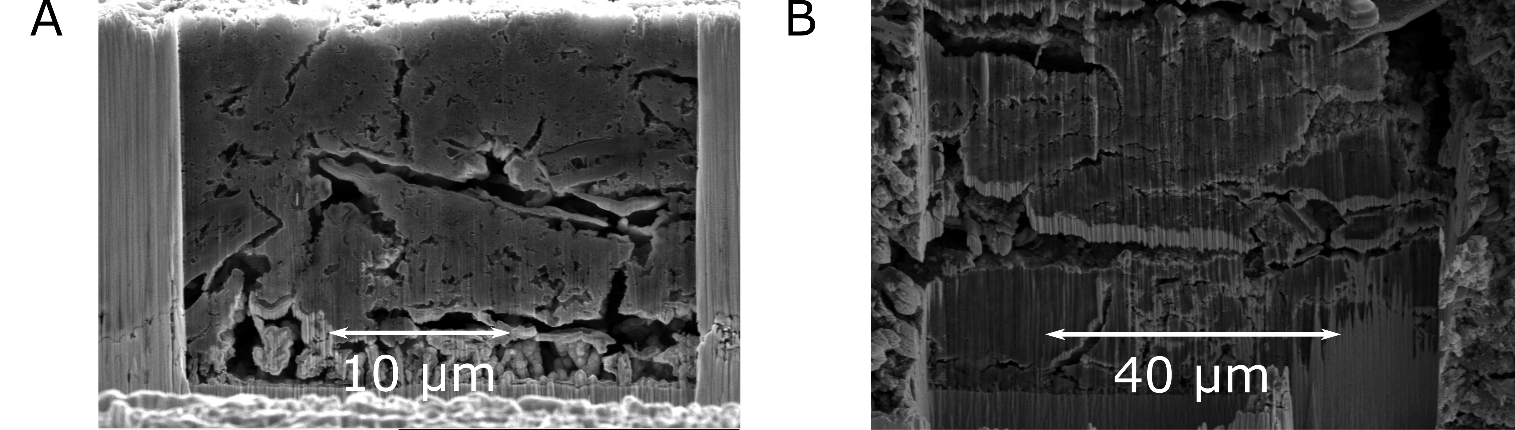


**Figure S10**. Cross-sectional SEM images of JUMP-1 (**A**) and JUMP-1(Li) (**B**) after 1000 charge-discharge cycles using 1 M LiTFSI in PC.

# SEM images of electrodes of the sodium system


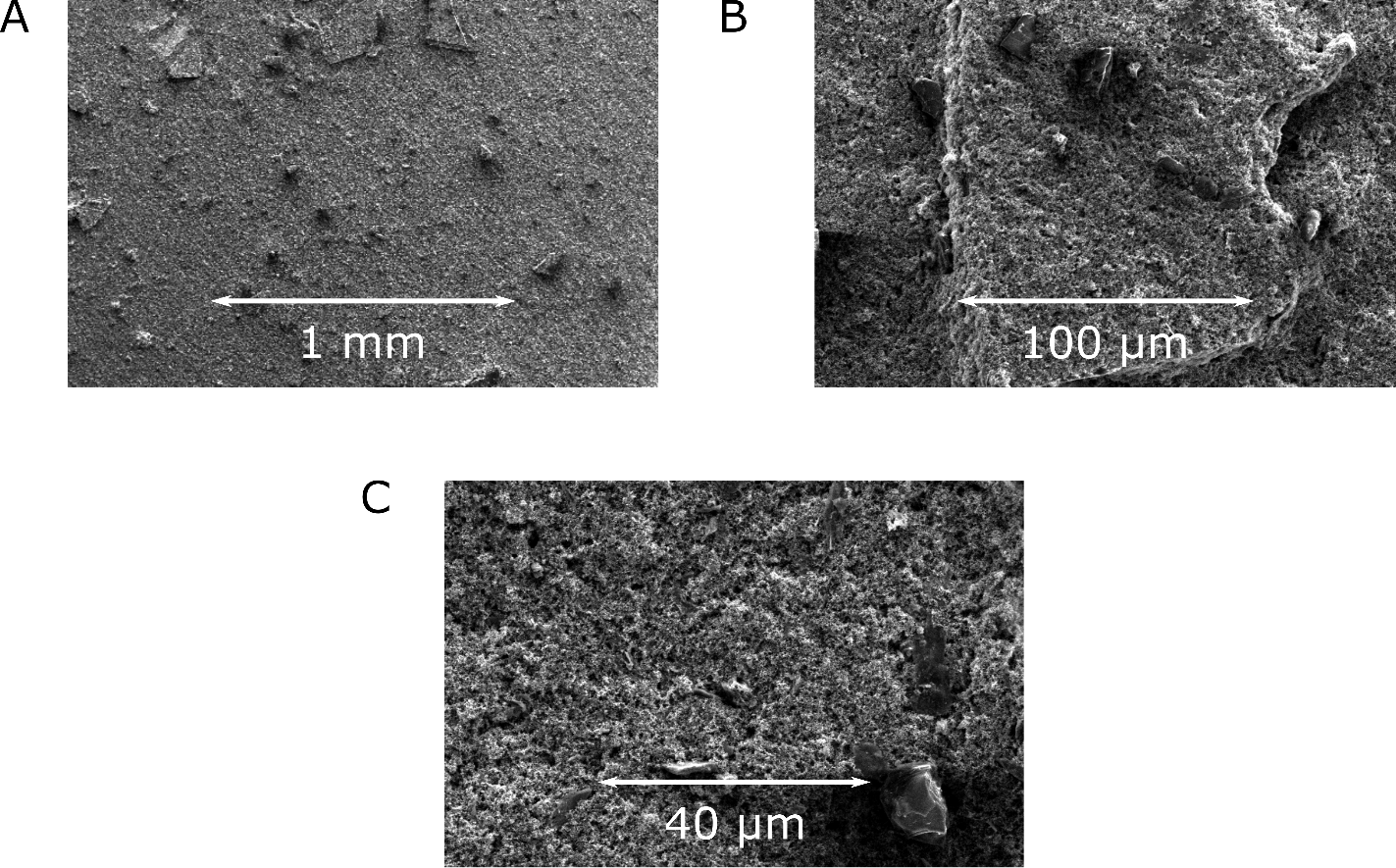


**Figure S11**. SEM images of the surface of the electrode with JUMP-1(Na) before electrochemical measurements shown with different magnifications.


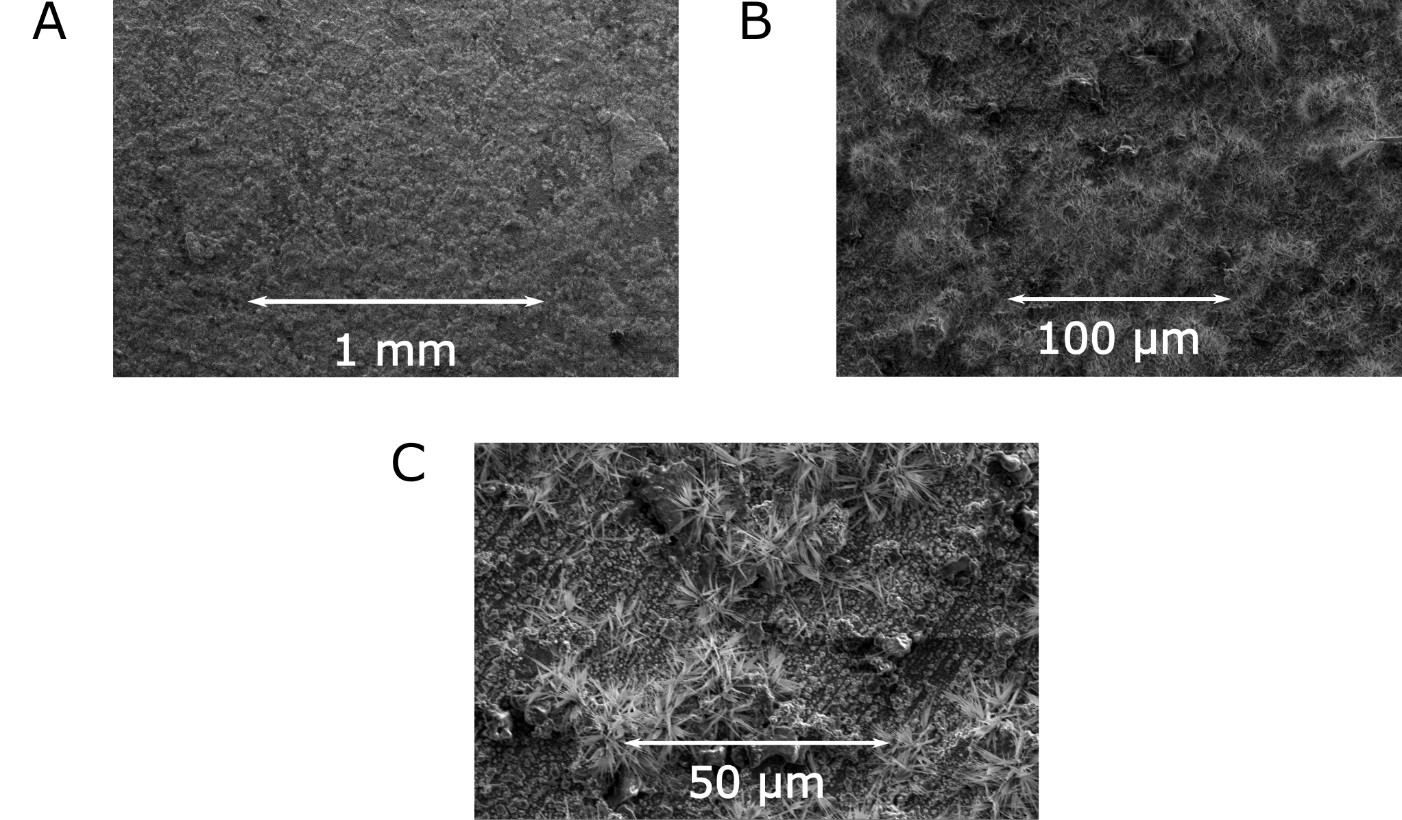


**Figure S12**. SEM images of the surface of the electrode with JUMP-1 after 1000 charge-discharge cycles using 1 M NaTFSI in PC shown with different magnifications.


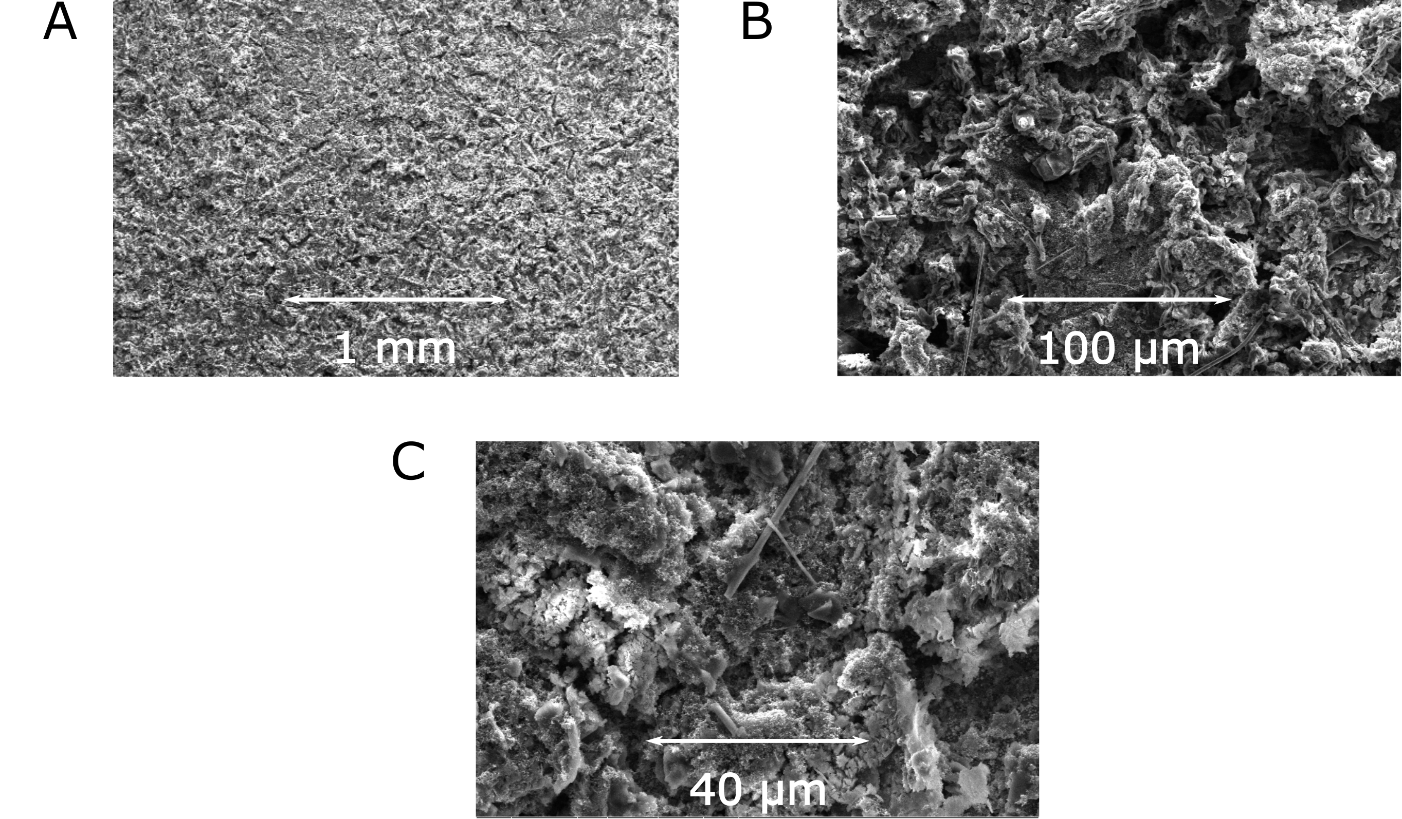


**Figure S13**. SEM images of the surface of the electrode with JUMP-1(Na) after 1000 charge-discharge cycles using 1 M NaTFSI in PC shown with different magnifications.

# Cross-sectional SEM scans of electrodes run in NaTFSI


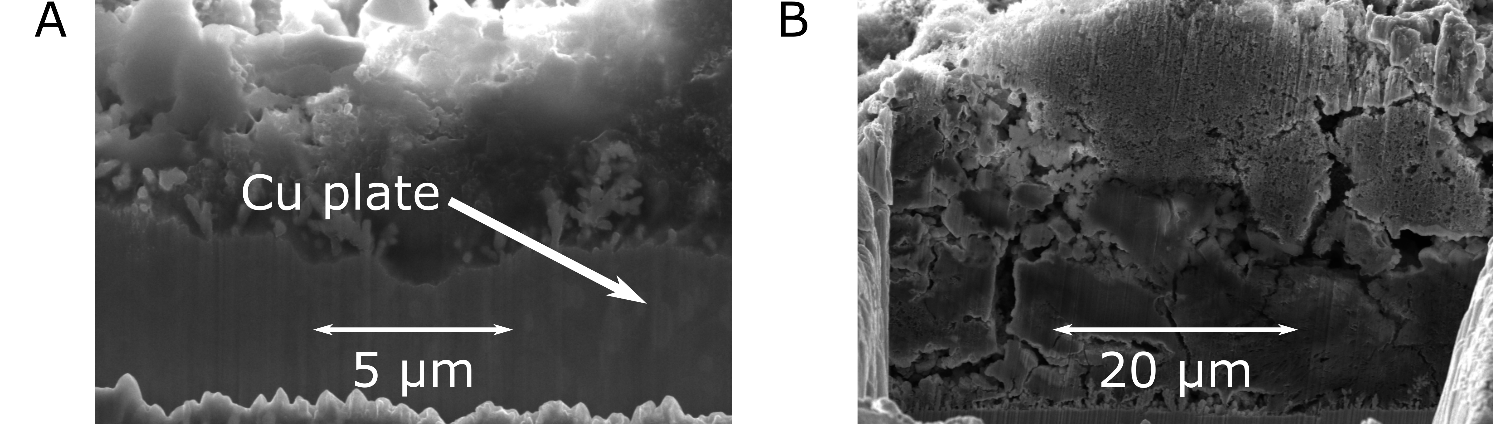


**Figure S14**. Cross-sectional SEM images of JUMP-1 (**A**) and JUMP-1(Na) (**B**) after 1000 charge- discharge cycles using 1 M NaTFSI in PC.
